# Entropy Correlation and Its Impacts on Data Aggregation in a Wireless Sensor Network

**DOI:** 10.3390/s18093118

**Published:** 2018-09-15

**Authors:** Nga Nguyen Thi Thanh, Khanh Nguyen Kim, Son Ngo Hong, Trung Ngo Lam

**Affiliations:** School of Information and Communication Technology, Hanoi University of Science and Technology, 11615 Hanoi, Vietnam; khanhnk@soict.hust.edu.vn (K.N.K.); sonnh@soict.hust.edu.vn (S.N.H.); trungnl@soict.hust.edu.vn (T.N.L.)

**Keywords:** entropy, correlation, distortion, data compression, representative node

## Abstract

A correlation characteristic has significant potential advantages for the development of efficient communication protocols in wireless sensor networks (WSNs). To exploit the correlation in WSNs, the correlation model is required. However, most of the present correlation models are linear and distance-dependent. This paper proposes a general distance-independent entropy correlation model based on the relation between joint entropy and the number of members in a group. This relation is estimated using entropy of individual members and entropy correlation coefficients of member pairs. The proposed model is then applied to evaluate two data aggregation schemes in WSNs including data compression and representative schemes. In the data compression scheme, some main routing strategies are compared and evaluated to find the most appropriate strategy. In the representative scheme, with the desired distortion requirement, a method to calculate the number of representative nodes and the selection of these nodes are proposed. The practical validations showed the effectiveness of the proposed correlation model and data reduction schemes.

## 1. Introduction

Wireless Sensor Networks (WSNs) are the collection of sensor nodes, which cooperatively monitor the surrounding environment over large physical areas. The latest achievements in the integration of micro-electro-mechanical systems and digital electronics with the development of wireless communications have enabled the wide deployment of WSNs. Sensor nodes in WSNs have been equipped with various sensing capabilities in space and time and higher processing capacities can satisfy requests from various modern applications. Due to low-cost, the small size, and no-replace battery powered characteristics of sensor nodes, energy conservation is commonly recognized as the key challenge in designing and operating the network.

In typical WSNs applications, sensors are normally deployed densely over the monitoring area to achieve satisfactory coverage [1]. As a result, there will be multiple sensors recording data from the same event in the sensing field, i.e., recorded data from these sensors will be correlated with each other. The existence of correlation characteristics has many significant potential advantages for the development of efficient communication protocols well-suited to the WSNs paradigm. For example, due to the correlation degree, data in a correlated region can be compressed with a high ratio to reduce communication load to save dissipated energy [2,3,4]. Moreover, with high enough correlation, it may not be necessary for every sensor node in a correlation group to transmit its data to the sink. Instead, a smaller number of sensor measurements (representation) might be adequate to communicate the event features to the sink within a certain reliability level [5].

To exploit the correlation characteristics in WSNs such as data compression and representative aggregations in the above example, correlated nodes must be clustered into correlated groups/regions. Therefore, it is necessary to develop a way to recognize the correlation among sensor nodes and build the correlation model. There have been many research efforts to study correlation in WSNs. In Reference [5], correlated nodes are supposed to observe the same source and the observed data is the sum of a correlated version of the source and the observed noise. The correlational model is distance-dependent and could be classified into four groups including Spherical, Power exponential, Rational quadratic, and Matérn. Among these groups, the power exponential correlational model is widely used, which is described in References [6,7,8]. Some papers also build correlational models in which the correlation coefficient is a function of distance among nodes [9,10]. In Reference [9], the correlation coefficient between two nodes is defined as an inverse proportion of their Euclidean distance. In Reference [10], the correlation coefficient is defined as an area ratio between the sensing intersection region of two nodes and the sensing region of a node, i.e., a function of the distance between nodes and sensing range.

Some other papers consider the correlation as the similarity of sensed data [11,12,13]. In Reference [11], to evaluate the difference of two-time series, magnitude dissimilarity (m-dissimilarity), and trend dissimilarity (t-dissimilarity) of two-time series are defined. In Reference [12], the dissimilarity is defined by using the Manhattan distance. To evaluate the sensed data similarity between two nodes, a sliding window is used to choose sensed data in both nodes with each dataset corresponding to a vector and the Manhattan distance of these two vectors expressing the similarity. The smaller the Manhattan distance, the more similarity between the two vectors. The Manhattan distance is also used to define the dissimilarity in Reference [13].

Some papers define the correlational model in different ways such as a linear predictive model [14], node weight [15], and a data density correlational degree [16]. In Reference [14], a set of sensor nodes is a correlational set if a reading at a node can be predicted by using a linear combination of readings from the other nodes. In Reference [15], the correlation of one node with its neighbors is evaluated by using spatially correlated weight. Spatially correlated weight is defined as the average spatial distance deviation between each node and its neighbors within a predefined communication range. In Reference [16], the data density correlation degree, which is a function of the average distance between one node and its neighborhood, is used to evaluate the correlation.

All the above models consider only the linear correlation between data and distance-dependence. To solve a more general correlational relation, entropy-based correlational models are considered [17,18,19]. In Reference [17], the joint entropy of a group of nodes is calculated by using a real dataset and then a distance-based joint entropy function is built by approximating the calculated joint entropy. An improvement is done in Reference [18]. Distance-based joint entropy correlation is also considered in Reference [19] for visual information in a wireless multimedia sensor network. In this research, the entropy correlation coefficient is defined as a function of the disparity between the two cameras.

**Figure 1:**
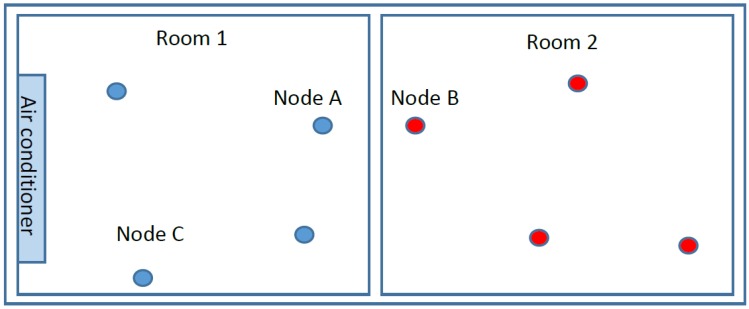
The layout of sensor nodes in an environment with two different conditions area.

The correlational models in the above studies are all based on distance among nodes. In addition, the distance information can be used to recognize the correlation easily. The smaller the distance between nodes, the higher the correlation they have. However, this assumption may not always be true because of some physical barriers among nodes. For example, in Figure 1, some sensor nodes are placed in two rooms next to each other in which room 1 is equipped with an air conditioner while room 2 is without an air conditioner. Node A and B are placed close to each other, but they are in different rooms with independent conditions. Therefore, their recorded data is also independent from each other. However, the sensed data in node A is correlated to node C because they are placed in the same room with the same conditions even though their distance is larger than the distance between node A and B. Therefore, it is necessary to establish a correlational model, which is distance-independent to the positions of sensor nodes. In Reference [20], when observing the readings over time of 54 sensors deployed at the Intel Berkeley Lab [21], it can be seen that the correlation of data may be independent of external factors such as sensor location and environmental conditions. Therefore, it is better to look at the information contained in the data itself rather than consider only attribute meta-data such as location and time.

The distance-independent, entropy-based correlation model is used in Reference [22]. However, instead of calculating directly from real data, the entropy correlation coefficient is chosen to be the Pearson linear correlation coefficient to reduce the computation complexity. On the other hand, it also reduces the generality of using entropy.

**Figure 2:**
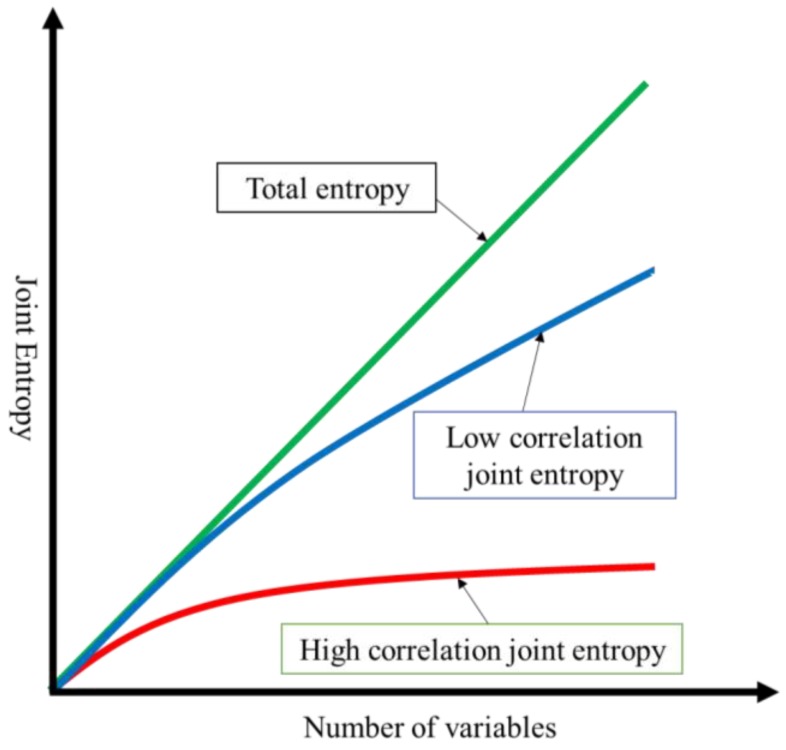
The relation between correlation and joint entropy with different correlational levels.

In Reference [23], with the expectation of looking at the data itself, the correlation is recognized by using the relation between the joint entropy of a set and the number of nodes in the set. The increase of the joint entropy of a group when one variable is added into the group is considered. If the added variable is highly correlated with variables in the group, i.e., it strongly depends on the variables in the group, the increasing of the joint entropy of the group by adding the variable is small. In other words, a small amount of additional information is needed to specify the added variable. Therefore, by considering the relation of joint entropy value with the number of variables in a group, it can be found that the increasing speed of the joint entropy value will gradually reduce and approach zero. Essentially, the joint entropy value moves to approach the “saturation” state when the number of considered variables increases. The more correlation among the nodes exists, the faster the joint entropy value moves to a “saturation” state. This phenomenon is described in Figure 2. The speed of approaching the “saturation” state could be specified to a correlational level.

In Reference [23], joint entropy is calculated from real data and then the joint entropy of a node set is approximated by an exponential function by the number of nodes in the set. However, this model can only be obtained when the correlational set has been established. There has not been an efficient way to recognize the correlation among nodes using the relation between joint entropy and the number of nodes in a set. To get the relation as described in Figure 2, in principle, the joint entropy values of all possible subgroups are calculated and an efficient process to check the relation between joint entropy and the number of nodes in all subgroups is required. That process is complicated with *O*(2*^n^*) complexity that requires vast of computation. This is not possible in practice.

In this paper, we focus on discovering and exploiting the general correlation in WSNs by using information entropy theory and by looking at the sensed data itself. To overcome these above difficulties on correlation recognition, we develop an approach to evaluate the joint entropy of a group as a function of the number of nodes in the group using entropy of individual nodes and the entropy correlation coefficient between pairs of nodes in the group. From this evaluation, the definition of a correlated region is proposed and the correlation-clustering scheme based on the proposed definition is presented. Using the proposed clustering scheme, sensor nodes are clustered into correlated region. Then data aggregation can be done to save energy. Thus, we consider of using data compression and representative aggregation in WSNs. The impacts of the proposed correlation model for the development of efficient data aggregations for WSNs including data compression and representative aggregation are considered. This paper is the correction and the extension of our previous studies in References [24,25].

The remainder of the paper is organized as follows. In Section 2, the estimation of the joint entropy of a group is presented. The validation of this estimation is also done in this section. Section 3 proposes a definition of the correlated region and then the correlation clustering scheme is described. In Section 4, the entropy correlation model is used to evaluate the main compression and aggregation schemes for WSNs. Lastly, the conclusion and future works are presented in Section 5.

## 2. Joint Entropy Estimation

### 2.1. Entropy Concept

To explore the correlational characteristics between the collected data, the concept of information entropy is used. In this section, at first, we review the concept of entropy and mutual information [26].

In information theory, the entropy of a random variable is a function that attempts to characterize the “unpredictability” or “uncertainty” of a random variable. If a random variable *X* takes on values in a set *X* = {*x*_1_, *x*_2_, …, *x_n_*} and is defined by a probability distribution *P*(*X*). Then the entropy *H*(*X*) of the random variable *X* is written below.

(1) $$\;H\left(X\right)=-\underset{x\in X}{\sum }P\left(x\right)logP\left(x\right)\;$$

The units of entropy are “bits” or “nats” depending on logp(x), which is based on base 2 logarithms or natural logarithms. In this paper, the base 2 logarithm is used instead of the natural logarithm and, hence, entropy is defined as the expected number of bits of information contained in each event. This has taken over all possibilities.

In the case of multi-random variables, the number of bits of information is calculated by joint entropy, which is the entropy of a joint probability distribution or a multi-valued random variable. For *n* random variables $X_{1},\;X_{2},\;\ldots ,\;X_{n}$, the joint entropy $H(X_{1},\;X_{2},\;\ldots ,\;X_{n})$ is defined by the equation below.

(2) $$\;H\left(X_{1},\ldots ,X_{n}\right)=-\underset{x_{1}\in X_{1}}{\sum }\ldots \underset{x_{n}\in X_{n}}{\sum }P\left(x_{1},\ldots ,x_{n}\right)\log_{2}P\left(x_{1},\ldots ,x_{n}\right)\;$$

where $x_{1},\;x_{2},\;\ldots ,\;x_{n}$ are particular values of $X_{1},\;X_{2},\;\ldots ,\;X_{n}$, respectively, and $P\left(x_{1},\ldots ,x_{n}\right)$ is the probability of these values occurring together.

Now let’s consider the case of two random variables *X* and *Y*. The relation between entropy and joint entropy is shown below.

(3)  H(X,Y)≤H(X)+H(Y) 

With equality if X and Y are independent. The above inequation shows that when the information covered by X fully comprised Y in its content, the joint entropy of two random variables equals the summation of the entropy of both variables. On the other hand, the joint entropy of these two variables is always smaller than the total entropy of these two variables. However, joint entropy alone cannot be used to evaluate the level of sharing information between pairs of random variables because the value of joint entropy depends on the single value of entropy of every variable.

Another metric used for measuring the mutual dependence between the two variables is mutual information, which measures the relationship between two random variables. In general, it measures how much information is communicated, on average, in one random variable about another. The formal definition of the mutual information of two random variables *X* and *Y* whose joint distribution is defined by P(X,Y) is given by the equation below.

(4) $$\;I\left(X,Y\right)=-\underset{x\in X}{\sum }\underset{y\in Y}{\sum }P\left(x,y\right)\log_{2}\frac{P\left(x,y\right)}{P\left(x\right)P\left(y\right)}\;$$

The relation between mutual information and entropy is given by the equation below.

(5)  I(X,Y)=H(X)+H(Y)−H(X,Y) 

It is difficult to compare the correlation level between two pairs of random variables using mutual information or joint entropy because their values depend on the entropy of each individual data in the pair. To overcome this problem, we use normalized measures of mutual information called the entropy correlation coefficient [27], which is given below.

(6) $$\;\rho \left(X,Y\right)=2\frac{I\left(X,Y\right)}{H\left(X\right)+H\left(Y\right)}=2-2\frac{H\left(X,Y\right)}{H\left(X\right)+H\left(Y\right)}\;$$

ρ(X,Y) is called the entropy correlation coefficient of the two random variables X and Y in the relation with mutual information I(X,Y) or joint entropy *H*(*X*,*Y*). The entropy correlational coefficient presents the comparative relationship of a pair of data independent to the value of individual entropy and, therefore, it can be used to compare the correlational level of two pairs of data.

From inequality (3), it can be found that the entropy correlation coefficient *ρ* varies from 0 to 1 (see Appendix A.1 for more explanation). The larger the value of *ρ*, the higher the correlation is. If *ρ* = 1, (in case *H*(*X*) = *H*(*Y*) = *H*(*X*, *Y*)), two sets of data totally depend on each other. If *ρ* = 0 (in case *H*(*X*, *Y*) = *H*(*X*) + *H*(*Y*)), they are independent.

### 2.2. Joint Entropy Estimation

Assume that there is a set of *N* data {*X*_1_, *X*_2_, …, *X_N_*} with the entropy of each data, *H*(*X_i_*), and entropy correlation coefficient, *ρ_ij_* = *ρ*(*X_i_*, *X_j_*) with any 1 ≤ *i* ≠ *j ≤ N* satisfies the following conditions.

(7) $$\;H_{min}\leq H\left(X_{i}\right)\leq H_{max}\;$$

(8) $$\;\rho_{min}\leq \rho_{ij}\leq \rho_{max}\;$$

The joint entropy is estimated based on the idea of hierarchical clustering [28].

#### 2.2.1. Joint Entropy Upper Bound

With a group that has only one node, the entropy of one node is limited by Equation (7).

(9) $$\;H_{1}=H\left(X_{i}\right)\leq k_{1}H_{max}\;$$

where *k*_1_ =1.

With a group of two nodes *X_i_* and *X_j_*, from the definition of entropy correlation coefficient in Equation (6), we have created the following formula.

(10) $$\;H_{2}=H\left(X_{i},X_{j}\right)=\frac{2-\rho \left(X_{i},X_{j}\right)}{2}\left(H\left(X_{i}\right)+H\left(X_{j}\right)\right)\;$$

In addition, $H\left(X_{i}\right),\;H\left(X_{j}\right)\leq H_{max}$ and $\rho \left(X_{i},X_{j}\right)=\;\rho_{ij}\geq \rho_{min}$.

Then

(11) $$\;H_{2}\leq \frac{2-\rho_{min}}{2}\left(2H_{max}\right)=\left(2-\rho_{min}\right)H_{max}\;$$

or $H_{2}\leq k_{2}H_{max}=bH_{max}$, where $k_{2}=b=2-\rho_{min}$

The coefficient *k*_2_ can also be rewritten below.

(12) $$\;k_{2}=\frac{b}{2}\cdot 2=\frac{b}{2}\left(k_{1}+1\right)\;$$

With a group of three nodes *X_i_*, *X_j_*, and *X_k_*, at first, the two nodes *X_i_* and *X_j_* are replaced by an equivalent node *X_ij_* with entropy $H\left(X_{ij}\right)$ equals to $H\left(X_{i},X_{j}\right)$ or we have $H\left(X_{ij}\right)=H\left(X_{i},X_{j}\right)\leq k_{2}H_{max}$. According to hierarchical clustering [19,28], the correlation coefficient between one cluster and another cluster can be obtained by the shortest correlation coefficient from any member of one cluster to any member of the other cluster. Therefore, the equation below is formed.

(13) $$\;\rho \left(X_{ij},X_{k}\right)=\min\left\{\rho \left(X_{i},X_{k}\right),\;\rho \left(X_{j},X_{k}\right)\}\right\}\geq \rho_{min}\;$$

Then,

(14) $$H_{3}=H\left(X_{i},\;X_{j},\;X_{k}\right)=H\left(X_{ij},\;X_{k}\right)=\frac{2-\rho \left(X_{ij},X_{k}\right)}{2}\left(H\left(X_{ij}\right)+H\left(X_{k}\right)\right)\leq \frac{2-\rho_{min}}{2}\left(k_{2}H_{max}+H_{max}\right)\;$$

Or

(15) $$\;H_{3}\leq \frac{b}{2}\left(k_{2}+1\right)H_{max}=k_{3}H_{max}\;$$

where $k_{3}=\frac{b}{2}\left(k_{2}+1\right)$.

Similarly, joint entropy *H_m_* of a group with *m* nodes could be considered as the joint entropy of a sub-cluster with *m-1* nodes and the remaining node. The entropy of the sub-cluster is joint entropy of *m-1* nodes and the entropy correlation coefficient between the sub-cluster and the main node is the greatest/shortest/average correlation coefficient from any member of the sub-cluster to the remaining node. Thus, the following formula is obtained.

(16) $$\;H_{m}\leq \frac{2-\rho_{min}}{2}\left(k_{m-1}H_{max}+H_{max}\right)=\frac{b}{2}\left(k_{m-1}+1\right)H_{max}=k_{m}H_{max}\;$$

where $k_{m}=\frac{b}{2}\left(k_{m-1}+1\right)$.

From the recurrence relation of *k_m_*, the general formula to calculate *k_m_* can be obtained as follows (*m* > 2).

(17) $$\;k_{m}=\;2{\left(\frac{b}{2}\right)}^{m-1}+{\left(\frac{b}{2}\right)}^{m-2}+\ldots +{\left(\frac{b}{2}\right)}^{2}+\frac{b}{2}\;$$

or in the more compact way (in case *b* ≠ 2):

(18) $$\;k_{m}=\frac{{\left(\frac{b}{2}\right)}^{m}-1}{\frac{b}{2}-1}+{\left(\frac{b}{2}\right)}^{m-1}-1\;$$

#### 2.2.2. Joint Entropy Lower Bound

The lower bound of the joint entropy of a group with *m* node could be determined in a similar way to the upper bound. In this case, the correlation coefficient between one cluster and another cluster can be obtained by the greatest correlation coefficient from any member of one cluster to any member of the other cluster. The results are below.

With a group that has only one node, we have formed the following equation.

(19) $$\;H_{1}=H\left(X_{i}\right)\geq l_{1}H_{min}\;$$

where *l*_1_ = 1.

With a group of *m* nodes (*m* ≥ *2*):

(20) $$\;H_{m}\geq l_{m}H_{min}\;$$

where $l_{m}=\frac{c}{2}\left(l_{m-1}+1\right)$ with $c=2-\rho_{max}$.

From the recurrent relation of *l_m_*, the general formula to calculate *l_m_* can be obtained as follows (*m* > 2):

(21) $$\;l_{m}=\;2{\left(\frac{c}{2}\right)}^{m-1}+{\left(\frac{c}{2}\right)}^{m-2}+\ldots +{\left(\frac{c}{2}\right)}^{2}+\frac{c}{2}\;$$

or in the more compact way (in case *c* ≠ 2):

(22) $$\;l_{m}=\frac{{\left(\frac{c}{2}\right)}^{m}-1}{\frac{c}{2}-1}+{\left(\frac{c}{2}\right)}^{m-1}-1\;$$

#### 2.2.3. Validation of Joint Entropy Estimation

To validate the proposed joint entropy estimation, at first, two special cases are considered. In the first case, all nodes completely depend on each other, i.e., all nodes measure the same information. In this case:

$H\left(X_{1}\right)=H\left(X_{2}\right)=\ldots =H\left(X_{m}\right)=H$, and $\rho_{ij}=1,\;\forall i,j=1,2,\ldots ,\;m;i\neq j$.

Thus $H_{min}=H_{max}=H$ and $\rho_{min}=\rho_{max}=1$, then $k_{m}=l_{m}=1$.

Using Equations (17) and (21), we have $H_{m}=H\left(X_{1},X_{2},\;\ldots ,X_{m}\right)=H$. These results show that the estimated joint entropy equal the actual joint entropy in this case.

In the second case, all nodes are completely independent of each other. In this case:

$\rho_{ij}=0,\;\forall i,j=1,2,\ldots ,\;m;i\neq j$, thus $\rho_{min}=\rho_{max}=0$. Then, $k_{m}=l_{m}=m$.

Using Equations (16) and (20), we have formed the following formula.

(23) $$\;mH_{min}\leq H_{m}=H\left(X_{1},\;X_{2},\ldots ,X_{m}\right)\leq mH_{max}\;$$

This inequality is true because in this case:

(24) $$\;H_{m}=H\left(X_{1},\;X_{2},\ldots ,X_{m}\right)=H\left(X_{1}\right)+H\left(X_{2}\right)+\ldots +H\left(X_{m}\right)\;$$

Moreover, to verify the above estimation of joint entropy in a practice, sample data supplied by the Intel Berkeley Research Lab [21] is used. The sample data was collected from 54 sensors deployed in the Intel Berkeley Research lab between 28 February, 2004 and 5 April, 2004. Mica2Dot sensors with weatherboards collected time stamped topology information along with humidity, temperature, light, and voltage values once every 30 seconds. Data were collected using the TinyDB in-network query processing system and were built on the TinyOS platform. In this paper, temperature data is considered an example for validation of the proposed estimation. A group of 11 nodes, which is named dataset 1, is chosen from 48 nodes with $\rho_{min}=0.6$, $H_{min}=2.16$, and $H_{max}=2.55$. The node selection algorithm will be presented in the next section. We choose 256 samples for each node to calculate entropy, joint entropy, and entropy correlation coefficients. The entropy of each node and the entropy correlation coefficient between each pair of nodes are shown in Table 1 and Table 2, respectively. From these entropies and the entropy correlation coefficients, the lower bound and upper bound of subsets from 11 nodes will be calculated alternatively. Additionally, the practical joint entropy of all considered subsets are calculated in comparison with an estimated lower bound and an upper bound. The results are shown in Table 3. It is found that the practical joint entropy of one subset is always between the lower bound and the upper bound. The above examples show the validity of the proposed estimation method.

## 3. Correlated Region and Correlation Clustering Algorithm

### 3.1. Estimated Joint Entropy and Correlation

As mentioned in Reference [23], correlated nodes share a large amount of information among them. Therefore, their joint entropy will not increase much when the number of nodes in the group increases. In other words, the joint entropy will go to a “saturation” state when the number of nodes increases. On the other hand, from Equations (16) and (20), it can be seen that the upper bound and the lower bound functions of joint entropy are the same with only an argument difference. If these differences are small enough, the difference between the upper bound and the lower bound is small. It means the real joint entropy value is similar with its upper or lower bound. Therefore, the upper/lower bound function is chosen to estimate the joint entropy of the group.

**Figure 3:**
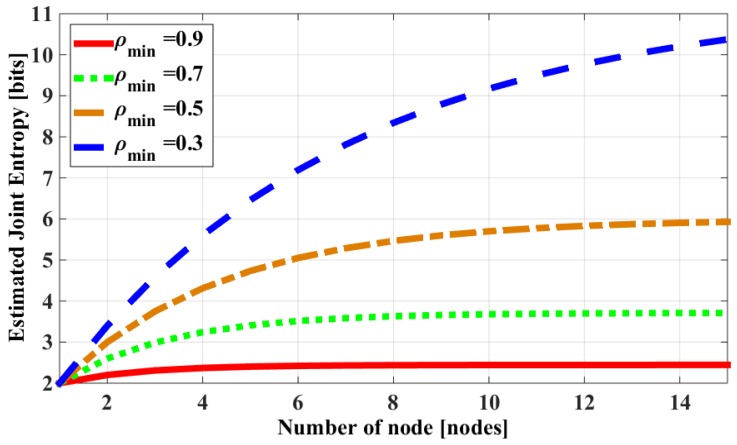
Estimated joint entropy with different values of entropy correlation coefficients using an upper bound function (with *H_max_* = 2 [bits]).

Among these two arguments, the entropy correlation coefficient has a strong effect on the shape of the function. Figure 3 shows the estimated joint entropy with different values of an entropy correlation coefficient.

It can be seen that, with a high enough value of an entropy correlation coefficient, the estimated joint entropy has the same characteristics as the calculated joint entropy of a correlation group. This means that the joint entropy will go to a “saturation” state when the number of nodes increases. The nodes with a higher correlation will approach the saturation state faster. From these results, it can be concluded that:

- It is acceptable to use a lower/upper bound function to estimate the joint entropy of a correlation group because they have similar characteristics of going to a “saturation” state when the number of nodes in the group increases.
- The entropy correlation coefficient of all pairs in the group can be represented by a correlation’s level of the group.

**Figure 4:**
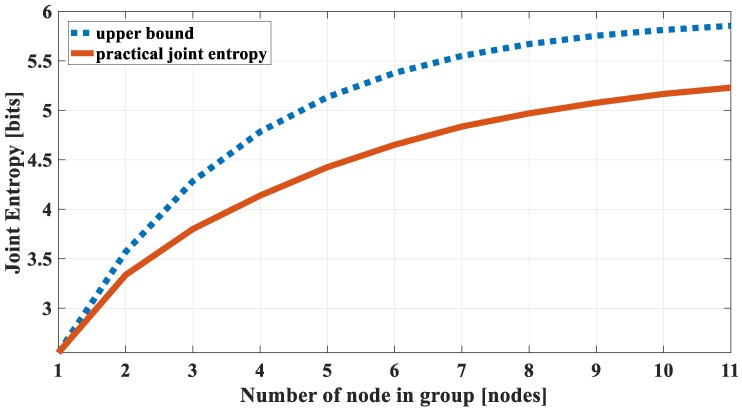
Estimated joint entropy (by the upper bound) and practical joint entropy of dataset 1.

When correlated nodes are grouped, it is expected that the joint entropy of the group is as small as possible. The worst case of joint entropy is its upper bound. Therefore, to evaluate the correlation of a group, the upper bound function should be used. Then the practical joint entropy will always be satisfied if its upper bound is already satisfied. Figure 4 shows the upper bound and practical joint entropy of the dataset 1 in the previous section. It can be seen that they have similar joint entropy characteristics of a correlational group. The difference between them is the difference between node entropy and the entropy correlation coefficient in practical data.

### 3.2. Correlated Region Definition

As mentioned in Reference [10], sensor nodes in the same correlated region record information of a single event in the sensor field, i.e., these sensed data are correlated with each other. Since the sensed data is taken from the same event, the number of bits to represent sensed data should be the same, i.e., the entropy of sensed data is similar. On the other hand, the entropy correlation coefficient of all pairs in this region is also similar.

Moreover, as shown in the last section, a group that has two properties such as similar entropy and a similar entropy correlation coefficient is a correlated group. Therefore, the correlated region can be defined as follows.

**Definition** **1.** *A correlated region with correlation level $\rho_{0}$ is a region in which the sensed data of all sensor nodes has the same entropy value and entropy correlation coefficient between all pairs of nodes are also the same and equal to $\rho_{0}$.*

(25) $$\;H_{0}=H\left(X_{1}\right)=H\left(X_{2}\right)=\ldots =H\left(X_{m}\right)\;$$

(26) $$\;\rho_{0}=\rho_{ij}=\rho \left(X_{i},X_{j}\right),\;\forall \;i\;\neq \;j\;$$

However, in practical cases, it is difficult to obtain the similarity between two entropies or the entropy correlation coefficient of pairs of nodes. Therefore, the correlated region can be defined in a more practical way, which is shown below.

**Definition** **2.** *A group of m nodes $\left\{X_{1},X_{2},\ldots ,\;X_{m}\right\}$ is in a correlated region with a correlation level $\rho_{0}$ if entropies of all member nodes vary in a very small range and entropy correlation coefficients between all pairs of nodes are larger than or equal to $\rho_{0}$.*

(27) $$\;H_{0}-\Delta H\leq H\left(X_{1}\right),H\left(X_{2}\right),\ldots ,H\left(X_{m}\right)\leq H_{0}\;$$

(28) $$\;\rho_{0}\leq \rho_{ij}=\rho \left(X_{i},X_{j}\right),\;\forall \;i\;\neq \;j$$

where Δ*H* is the entropy variation range. *H*_0_ is called “base entropy” and *ρ*_0_ is called “correlation level” of the data collected in the region. The higher the correlation level, the greater the amount of correlation of the collected data is in this region. In this paper, if a region has $\rho_{0}\geq 0.5$, we call it is a highly correlated region.

With this definition, we can estimate the joint entropy of a group *m* nodes $\left\{X_{1},X_{2},\ldots ,\;X_{m}\right\}$ by the following equation.

(29) $$\;H_{m}=H\left(X_{1},X_{2},\;\ldots ,\;X_{m}\right)=k_{m}H_{0}\;$$

in which *k_m_* is calculated by using Equation (17) or Equation (18) with *b* = 2 − *ρ*_0_. This equation is called the entropy correlation model. It will be used for analysis and evaluation of the impact of the proposed correlation definition in the next section.

One problem that arises with Definition 2 is how the entropy variation range Δ*H* affects the precision of the joint entropy value. To answer this question, we consider the variation of the joint entropy value corresponding to the entropy variation. For simplicity, it is supposed that $\rho_{0}=\rho_{ij}=\rho \left(X_{i},X_{j}\right),\;\forall \;i\;\neq \;j$. From Definition 2, the real joint entropy value *H_rm_* of the group including *m* nodes $\left\{X_{1},X_{2},\ldots ,\;X_{m}\right\}$ is limited by the following inequation.

(30) $$\;k_{m}\left(H_{0}-\Delta H\right)\leq H_{rm}\leq k_{m}H_{0}\;$$

Then,

(31) $$\;\frac{k_{m}\left(H_{0}-\Delta H\right)}{k_{m}H_{0}}\leq \frac{H_{rm}}{k_{m}H_{0}}\leq 1\;$$

Or

(32) $$\;1-\frac{\Delta H}{H_{0}}\leq \frac{H_{rm}}{H_{m}}\leq 1\;$$

The above equation describes the effect of an entropy variation range Δ*H* to the difference between the real joint entropy value and the estimated joint entropy value. The smaller the entropy variation range Δ*H* is, the smaller the difference is. Therefore, the entropy variation range Δ*H* is chosen by depending on the requirement of the precision *e* (%) of the estimated joint entropy value.

(33) $$\;1-\frac{\Delta H}{H_{0}}\leq \frac{H_{rm}}{H_{m}}\leq e\left(\%\right)\Rightarrow \frac{\Delta H}{H_{0}}\leq \left(100-e\right)\left(\%\right)\;$$

In this paper, we choose the error between the real and the estimated joint entropy value, which is *e* = 85%. Then $\Delta H\leq 15\%H_{0}$.

### 3.3. Correlation Clustering Algorithm

Using the definition of the correlated region, a sensor field can be divided into correlated regions with a specified base entropy and a correlational level. The clustering algorithm is described in Algorithm 1. At first, an entropy range and a correlation level are chosen. Next, nodes with their entropy values in the entropy range are selected into a group. Then, the entropy correlation coefficient of all pairs in the group is calculated and a node with the highest number of pairs that satisfied the correlational level is chosen as a core node. Next, nodes in the group that the correlation coefficients between them and the core node are smaller than the correlation level will be removed from the group. After that, the process of removing a node with the highest number of pairs that do not satisfy correlation level is repeated until all pairs in the group satisfy the correlation level.

**Algorithm 1**: Correlation-based Clustering Algorithm1. **BEGIN** 2. **FOR** each node *X_i_* in the network 3.  **Calculate** entropy *H*(*X_i_*) 4. **ENDFOR** 5. **FOR** each node *X_i_* in the network 6.  **FOR** each node *X_j_* in the network and *X_j_* ≠ *X_i_* 7.    **Calculate**
$\rho \left(X_{i},\;X_{j}\right)$
8.  **ENDFOR** 9. **ENDFOR** 10. **REPEAT** 11.  **Choose**
*H*_0_, *ρ*_0_, Δ*H*; (*) 12.  **Initialize** new group *G* = *Φ*. 13.  **FOR** each node *X_i_* in the network and not belonging to any group 14.    **IF**
$H_{0}\leq H\left(X_{i}\right)\leq H_{0}+\Delta H$ 15.      **Add**
*X_i_* into *G* 16.    **ENDIF** 17.  **ENDFOR** 18.  **FOR** each node *X_i_* in *G* 19.    **Calculate**
*B*(*X_i_*) = number of nodes *X_j_* that $\rho \left(X_{i},\;X_{j}\right)\geq \rho_{0}$ 20.  **ENDFOR** 21.  *X*_0_ = ***arg*max**{*B*(*X_j_*), *X_j_∈G*} 22.  **FOR** each node *X_i_* in *G* 23.    **IF**
$\rho \left(X_{i},X_{0}\right)<\rho_{0}$ 24.      **Remove**
*X_i_* from *G* 25.    **ENDIF** 26.  **ENDFOR** 27.  **REPEAT** 28.    **FOR** each node *X_i_* in *G* 29.      **Calculate**
*C*(*X_i_*) = number of nodes *X_j_* that $\rho \left(X_{i},\;X_{j}\right)<\rho_{0}$ 30.    **ENDFOR** 31.    **FOR** each node *X_i_* in *G* 32.      **IF** 0 < *C*(*X_i_*) = ***max***{*C*(*X_j_*), *X_j_∈G*} 33.        **Remove**
*X_i_* from *G* (**) 34.      **ENDIF** 35.    **ENDFOR** 36.  **UNTIL *max***{*C*(*X_j_*), *X_j_∈G*}=*0* 37. **UNTIL** all nodes are grouped 38. **END**

In the step (*) of the algorithm, the base entropy and correlation level are chosen so that they can cover all possible values of entropy and the entropy correlation coefficient in the network. The value of the entropy correlation coefficient should be chosen from high to low. In the step (**) of the algorithm, if there is more than one node that satisfies the condition *0 < C*(*X_i_*) = max{*C*(*X_j_*), *X_j_∈G*}, the node that has a maximum entropy value will be removed.

It should be noted that the entropy of each individual node’s data and entropy correlation coefficients of each data pairs must be known beforehand to implement the clustering. Therefore, a data acquisition period is required at the beginning to collect enough data. The calculation of entropy values and entropy correlation coefficients must be done before the clustering algorithm and applications of correlation clustering.

Using the proposed correlation clustering algorithm, the sensor nodes in Section 2.2 are clustered similarly to Table 4. The network is divided into four groups. The first group includes 11 nodes with *ρ*_0_ = 0.6. The second group includes six nodes with *ρ*_0_ = 0.6 but in a different entropy value range. The third group includes eight nodes with *ρ*_0_ = 0.5*.* All other nodes belong to the last group, which is weakly correlated. The first group is used as an example of a correlation group in Section 2.

**Figure 5:**
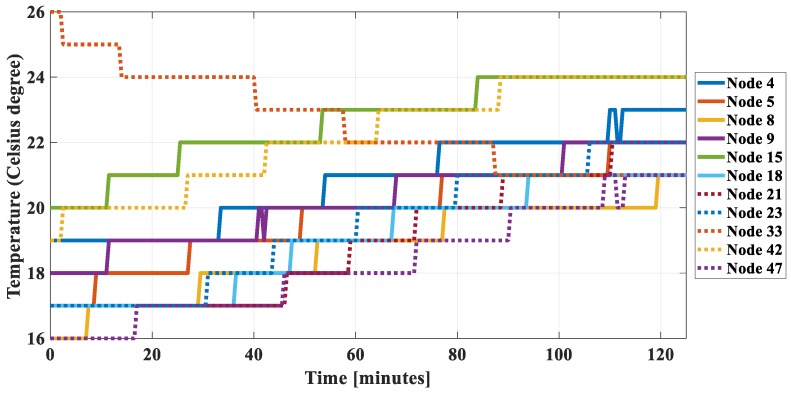
Temperature data measured at 11 nodes in the dataset 1.

In addition, to calculate joint entropy to show the correlation among nodes in the established correlation group, Figure 5 shows the practical sensed data of 11 nodes in the first group. It can be found that data of all nodes except node 33 are quite similar, i.e., they vary with time in the same way. Data of node 33 looks different from the others, but its negative varies similarly to the other. Thus, they are all correlated with each other.

### 3.4. Validation

To validate the practical Definition 2 in comparison to the theoretical Definition 1, we complete a comparison between the estimated joint entropy using Equation (29) and practical calculation of the selected group by Definition 2. The result is shown in Figure 4. It can be seen that they have similar shapes, but there is a difference between estimated values and practical values. As explained in the previous section, the difference is caused by the dissimilarity in entropy values and correlation coefficients of nodes in the practical environment. However, the most important thing is the correlational characteristics (joint entropy values go to a “saturation” state when the number of nodes increases) has been preserved.

**Figure 6:**
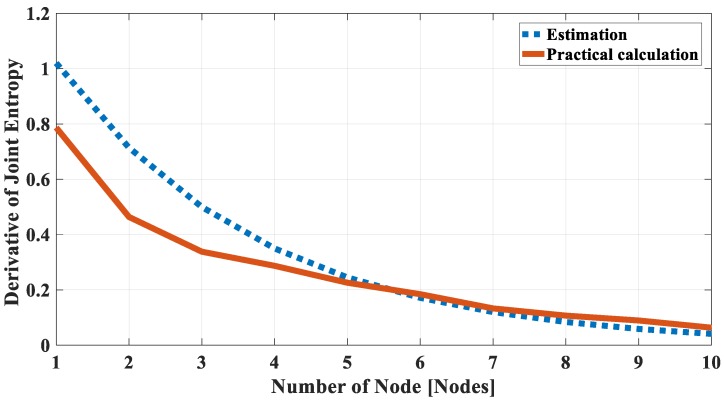
The derivative of estimated joint entropy and calculated joint entropy of the dataset 1.

To check the correlational characteristics, the derivative of the joint entropy function by the node’s number in a group ($\Delta H_{n}/\Delta n=H_{n}-H_{n-1}$) is calculated for both the estimated and practical joint entropy values, which is shown in Figure 6. It is found that the derivatives are similar and reduced when the number of nodes increases for both cases. It means that the joint entropy values go to a “saturation” state when the number of node increases. The correlational characteristic is preserved, and it is possible to use the estimated joint entropy model by Equation (29) to estimate the joint entropy of a correlated group established by using Definition 2.

For complexity evaluation, in case of using the relation between joint entropy and the number of nodes in the group by direct calculation, the joint entropy values of all possible subgroups must be calculated. Then the relations between joint entropy and the number of nodes in all subgroups are checked. With a group with n nodes, there are 2*^n^* − 1 different subgroups and the complexity of this direct calculation is *O*(2*^n^*). However, by using the proposed clustering algorithm, we only have to calculate the entropy of individual nodes and an entropy correlation coefficient of all pairs in the considered group. The complexity for this calculation is *O*(*n*^2^). Then using the proposed clustering algorithm with *O*(*n*^3^) complexity, correlation subgroups can be established. Thus, the complexity of the proposed calculation for correlation recognition is only O(*n*^3^), which is much smaller than *O*(2*^n^*) of a direct calculation.

## 4. Data Aggregations Using Entropy Correlation

According to the aggregation strategies, data level aggregation methods are divided into three types: in-network query type, data compression type, and representative type [16]. Correlation is appropriated with the data compression type and the representative type. This section considers the applications of the proposed entropy correlation model into data aggregation in WSNs including compression aggregation and representative aggregation. In this paper, it is assumed that correlated nodes are geographically close to each other. Thus, a correlated region may correspond to a geographical region.

### 4.1. Compression Aggregation

#### 4.1.1. Comparison of Compression Schemes

Using the correlation-based clustering algorithm, correlation nodes are grouped into a correlated region and data compression can be done to reduce the amount of data transfer in the network to save energy. There are many compression techniques that can be applied in a wireless sensor network [2,3,4].

In Reference [17], the impact of correlation to data compression has been analyzed using its distance-based correlation model. In this paper, the impact of correlation to compression will be evaluated using the same framework in Reference [17] but, instead, using our proposed correlation model.

**Figure 7:**
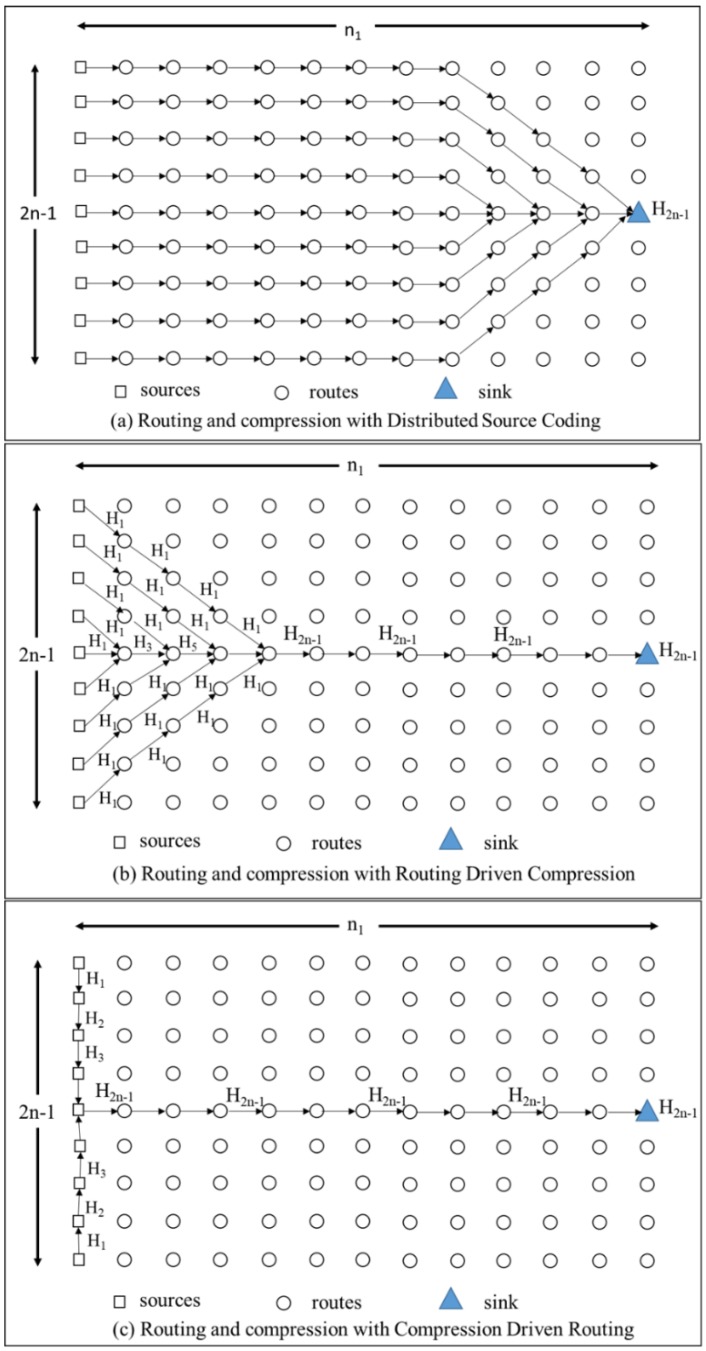
Routing paths for three schemes: (**a**) DSC, (**b**) RDC, and (**c**) CDR in Reference [17].

To choose the most appropriate lossless compression approach like Reference [17], we will evaluate three main qualitatively different routing schemes: Distributed Source Coding [29], Routing Driven Compression [30,31], and Compression Driven Routing [32]. We consider the arrangement of sensor nodes in a grid where only (2*n* − 1) nodes in the first column are the sources. There are *n*_1_ hops on the shortest path between the sources and the sink [17]. The paths taken by data and the intermediate aggregation of three considered schemes are shown in Figure 7.

In Distributed Source Coding (DSC), the sensor nodes know about their correlations and they can compress data to avoid transmitting redundant information. In this case, ideally, each source will send exactly the right amount of uncorrelated data to the sink along the shortest path possible without the need for intermediate compression. The energy consumption for this scheme (*E_DSC_*) is calculated as:

(34) $$\;E_{DSC}=n_{1}H\left(X_{1}\right)+n_{1}H\left(X_{2}|X_{1}\right)+\ldots +n_{1}H\left(X_{2n-1}|X_{1},X_{2},\;\ldots X_{2n-2}\right)=n_{1}H_{2n-1}\;$$

In Routing Driven Compression (RDC), the sensor nodes do not know about their correlations and send data along the shortest paths to the sink while allowing for opportunistic compression wherever the paths overlap. The energy consumption for this scheme (*E_RDC_*) can be derived by the equation below.

(35) $$\;E_{RDC}=\left(n_{1}-n\right)H_{2n-1}+2H_{1}\overset{n-1}{\underset{i=1}{\sum }}i+\overset{n-1}{\underset{j=1}{\sum }}H_{2j-1}\;$$

In Compression Driven Routing (CDR), nodes have no knowledge of the correlation, but the data is compressed close to the sources and initially routed for maximum possible compression at each hop. The energy consumption for this scheme (*E_CDR_*) is shown below.

(36) $$\;E_{CDR}=n_{1}H_{2n-1}+2\overset{n}{\underset{i=1}{\sum }}H_{i}\;$$

**Figure 8:**
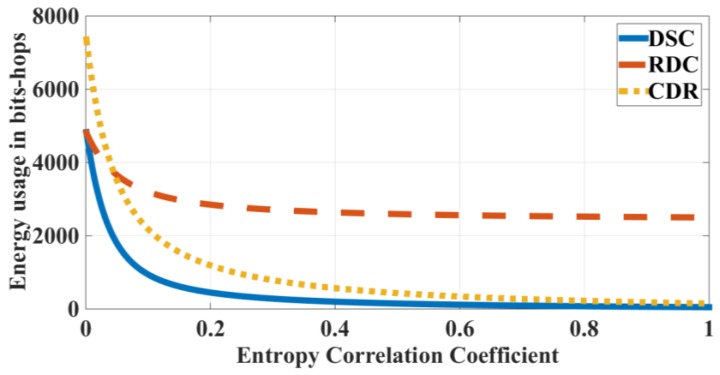
Energy expenditures for the DSC, RDC, and CDR schemes respectively to entropy correlation coefficients.

Using the estimated joint entropy model in Equation (29) for the above expressions, we can quantify the performance of each scheme. Figure 8 plots the energy consumption for the Distributed Source Coding, Routing Driven Compression, and Compression Driven Routing schemes as a function of the entropy correlation coefficient (with *n* = *n*_1_ = 50, *H*_0_ = 1 bit).

It can be seen that the Distributed Source Coding scheme has the lowest energy consumption because the number of transmitted bits is the smallest among lossless compression schemes. The higher the correlation level, the smaller the energy usage is. For the Routing Driven Compression scheme, the correlation level does not affect much of the energy usage of the scheme. For the Compression Driven Routing scheme, the energy usage is high with a small correlation level, but it reduces considerably when the correlation level increases. The Compression Driven Routing scheme approaches Distributed Source Coding in a high correlation area. The above results are similar to the results in Reference [17], which used a different correlation model.

From this result, the Distributed Source Coding and Compression Driven Routing are appropriate for the wireless sensor network with high correlation characteristics of the environment. However, the Distributed Source Coding scheme is quite difficult to implement when compared to the Compression Driven Routing, which can be implemented easily by local compression. Therefore, Compression Driven Routing is highly recommended to be the compression approach for a wireless sensor network with high correlation characteristics.

#### 4.1.2. Compression Based Routing Scheme in a Correlated Region

In the previous sections, we have proposed a clustering scheme to divide a wireless sensor network into correlated regions with a specified correlational level. In addition, the comparison among several compression-based routing schemes has been done and it is shown that CDR is a suitable scheme. However, according to our proposed definition of the correlated region, all nodes in the same correlated region are correlated with the same correlational level. This leads to the existence of many equivalent CDR paths with a similar maximum compression possibility. Therefore, to get the optimal routing scheme in a correlated region, it is necessary to consider the shortest path routing in combination with CDR. In this paper, it is assumed that correlated nodes are geographically close to each other. Thus, the idea of finding the optimal compression-based routing in each correlated region is to use distance-based clustering with data sending along the shortest paths to cluster head in each cluster. Similar to Reference [17], we will analyze the energy consumption of clustering-based routing schemes for 1-D, 2-D, and general topology networks when compression is performed using our proposed correlation model. Two compression situations are considered including compression at intermediate nodes on the short path tree (SPT) and only at the cluster heads.

#### 4.1.3. 1-D Analysis

**Figure 9:**
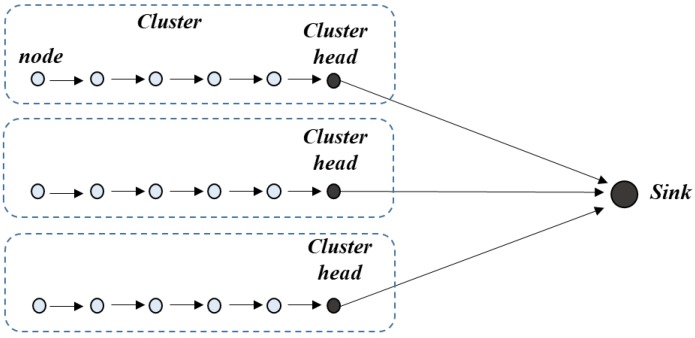
Routing pattern of the 1-D network.

Considering *N* source nodes linearly located with unit spacing on one side of a 2-D grid of nodes and the sink on the other side. These source nodes are divided into *N*/*s* clusters in which each cluster consists of *s* nodes. The cluster head for each cluster is supposed to be located at the end of each cluster. The routing pattern is shown in Figure 9.

As mentioned in the previous subsection, we will consider two types of compression performances. With the first type, the data is compressed sequentially from one end to the cluster head end. In the second type, the compression is done only at the cluster head. The cluster head then sends the compressed data along the shortest path involving *P* hops to the sink. The total bit-hop cost for this routing scheme is shown below.

(37) $$\;E_{s}\left(\rho_{0}\right)=\frac{N}{s}\left(E_{1in}+E_{1ex}\right)\;$$

where *E*_1*in*_ is the bit-hop cost within each cluster and *E*_1*ex*_ is a bit-hop cost for each cluster to send compressed information to the sink.

a. Sequential Compression along a Short Path Tree to the Cluster Head

In this case, at each hop, a node receives data from its previous hop, compresses them with its own data, and transmits compressed data to the next hop. The total bit-hop cost could be obtained as follows (detailed proof is in Appendix A.2).

(38) $$\;E_{s}\left(\rho_{0}\right)=\frac{N}{s}\left(\frac{b\cdot s}{2-b}+1+\left(P-\frac{2}{2-b}\right)k_{s}\right)H_{0}\;$$

where the coefficient *k_s_*, according to Equation (18), is calculated by using the formula below.

(39) $$\;k_{s}=\frac{{\left(\frac{b}{2}\right)}^{s}-1}{\frac{b}{2}-1}+{\left(\frac{b}{2}\right)}^{s-1}-1\;$$

with *b* = 2 − *ρ*_0_.

From Equation (38), it can be found that the optimal value of cluster size depends on the joint entropy coefficient *ρ*_0_ and number of hops *P* to the sink.

**Figure 10:**
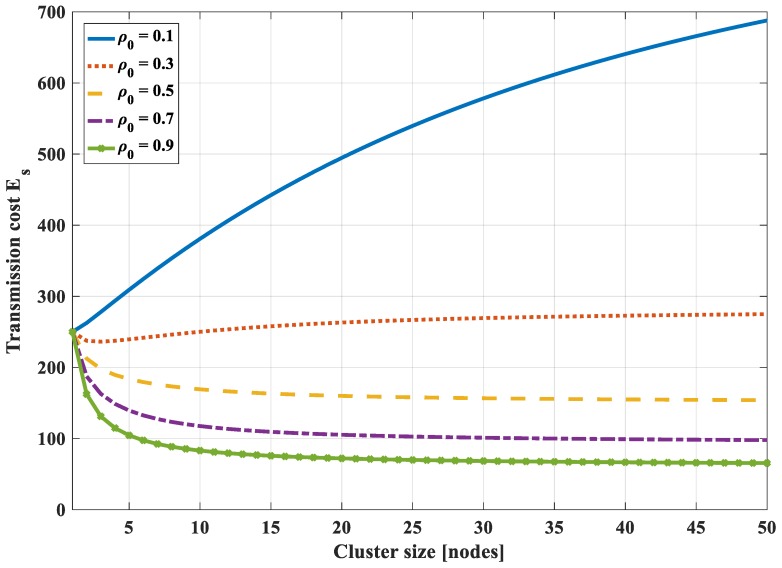
Total bit-hop cost *E_s_* respectively to cluster size with different values of an entropy correlation coefficient in the case of 1-D with compression along SPT to the cluster head.

It is difficult to find the formula for the optimal value of the cluster size. Instead, we will consider the total bit-hop cost *E_s_*, respectively, to cluster sizes with some different values of joint entropy coefficient *ρ*_0_. Figure 10 shows the total bit-hop cost *E_s_*, respectively, to cluster size with different values of *ρ*_0_ (in this case *N* = 50, *P* = 5, *H*_0_ = 1). It is found that, in the case of very low correlation (*ρ*_0_ = 0.1), the optimal number of cluster size is *s_opt_* = 1. It means that, in this case, clustering does not bring any efficiency in energy saving. All nodes will communicate directly with the sink instead of through an intermediate node.

In case of high correlation (*ρ*_0_ ≥ 0.5), the optimum number of cluster sizes is *s_opt_* = *N*, i.e., it is not necessary to cluster the correlated region into smaller subsets. The data is compressed sequentially from the node end to the cluster head end with all nodes in the cluster. The head will compress data and get sent to the sink. Because of high correlation, the more the number of nodes, the higher the compression rate of data. In case the entropy correlation coefficient is low (*ρ*_0_ = 0.3), there exists an optimum number of the cluster size but a small value. The correlated region should be clustered into some smaller subsets to optimize transmission cost. However, with this low correlation and a small number of correlated nodes, the effectiveness of the correlation characteristic is not significant.

The optimal value of cluster size also depends on the number of hops (*P*) to the sink. If *P* is large, the energy dissipation for transmitting data from the cluster head to the sink is larger than energy dissipation in the other tasks. The amount of data sent to the sink should be reduced. Moreover, the compression of highly correlated data is more efficient if the number of the dataset is higher. Thus, the number of nodes in each cluster should be increased, i.e., the number of the clusters should be reduced or may even not be necessary to divide the correlated region into smaller subsets.

From the above analysis, it can be concluded that, with the highly correlated region (*ρ*_0_ ≥ 0.5), it is not necessary to divide a correlation cluster into smaller groups. The whole region would be a cluster and all nodes will send data to the cluster head. The cluster head will compress the data and will send the data to the sink.

b. Compression at a Cluster Head Only

In this case, each node receives data from the previous hop and transfers them with its own data to the next hop without compression. The cluster head receives data from all nodes in the cluster, compresses them, and sends the data to the sink. Therefore, the total bit-hop cost could be obtained as follows (detailed proof is in Appendix A.3).

(40) $$\;E_{s}\left(\rho_{0}\right)=N\left(\frac{s-1}{2}+\frac{P\cdot k_{s}}{s}\right)H_{0}\;$$

where the coefficient *k_s_* is calculated below.

$$\;k_{s}=\frac{{\left(\frac{b}{2}\right)}^{s}-1}{\frac{b}{2}-1}+{\left(\frac{b}{2}\right)}^{s-1}-1\;$$

with *b* = 2 − *ρ*_0_.

**Figure 11:**
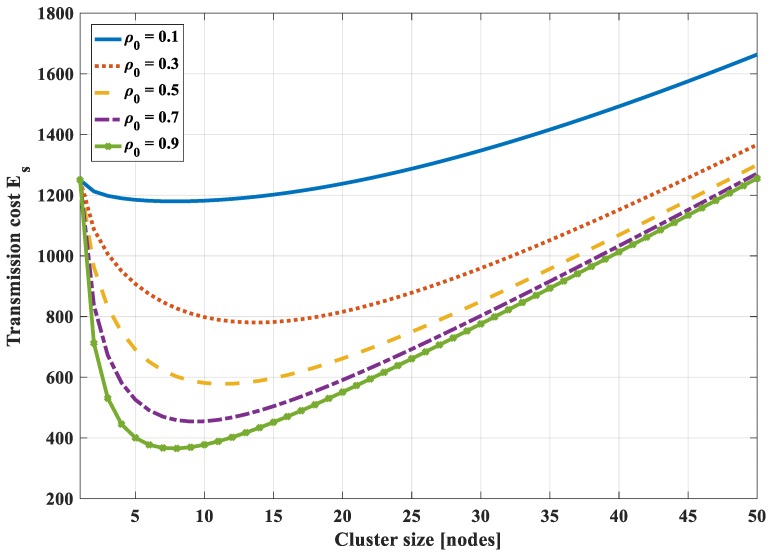
Total bit-hop cost *E_s_*, respectively, to cluster size with different values of an entropy correlation coefficient in the case of 1-D with compression at a cluster head only.

Figure 11 shows the total bit-hop cost *E_s_*, respectively, to cluster size with different values of *ρ*_0_ (in this case *N* = 50, *P*= 30, *H*_0_ = 1). It is found that there exists an optimal value of cluster size that is not at the two ends of the graph (*s* ≠ 1 and *s* ≠ *N*). However, this optimal value depends on the correlation coefficient. With a high correlation value (*ρ*_0_ ≥ 0.5), the optimal cluster size value reduces when the correlation increases. In addition, it is found that the optimal value strongly depends on the number of hops (*P*). The higher the value of the number of hops, the higher the value of the optimal cluster size value. This is because the energy dissipation for transmitting data from the cluster head to the sink becomes dominant in comparison to the other tasks. The amount of data sent to the sink should be reduced. Moreover, the compression of highly correlated data is more efficient if the number of the dataset is higher. Thus, the number of nodes in each cluster should be increased, i.e., the cluster size should be higher.

#### 4.1.4. 2-D Analysis

**Figure 12:**
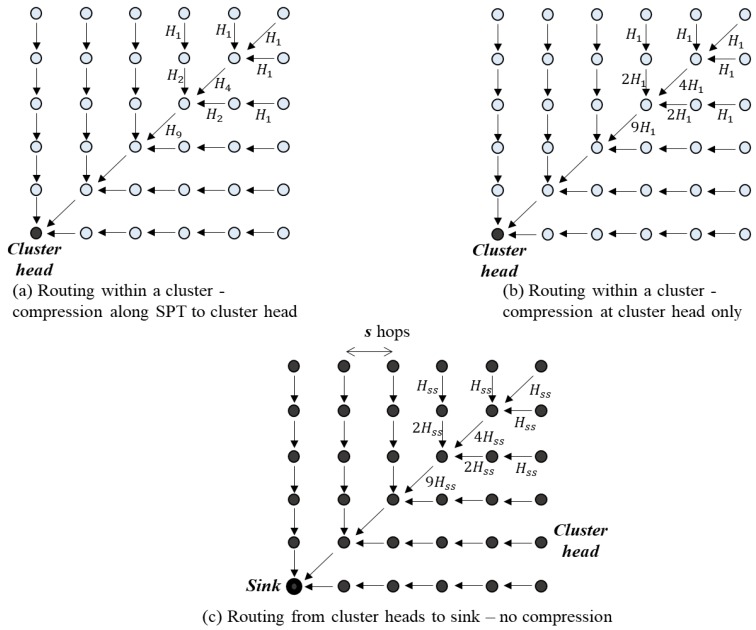
Routing pattern of the 2-D network in Reference [17].

Consider a 2-D network with *N* = *n^2^* nodes located on an *n × n* unit grid. The network nodes are divided into clusters of size *s × s* with *s*^2^ nodes. The routing patterns within a cluster and from cluster heads to sinks are demonstrated in Figure 12. In each cluster, data is transmitted to cluster heads with compression at each hop (Figure 12a) or with compression at the cluster head only (Figure 12b). Data from cluster heads is transmitted to sink without any compression along the transmission path (Figure 12c). The total bit-hop cost for this routing scheme is shown below.

(41) $$\;E_{s}\left(\rho_{0}\right)=E_{2in}+E_{2ex}\;$$

where *E*_2*in*_ is a total bit-hop cost within clusters and *E*_2*ex*_ is the total bit-hop cost for clusters to send compressed information to the sink.

a. Opportunistic Compression along Short Path Tree to the Cluster Head

In this case, at each cluster, a node receives data from the previous hop, compresses them with its own data, and transmits to the next hop to reach the cluster head, which is shown in Figure 12a. The cluster head compresses all received data and sends the data to the sink without any compression in the transfer path, which is shown in Figure 12c. The total bit-hop cost can be obtained by the formula below (detailed proof is in Appendix A.4).

(42) $$\;E_{s}\left(\rho_{0}\right)=\left[{\left(\frac{n}{s}\right)}^{2}\overset{s-1}{\underset{i=1}{\sum }}\left(2\left(s-i\right)k_{i}+k_{i^{2}}\right)+\frac{n}{6}\left(\frac{n}{s}-1\right)\left(4\frac{n}{s}+1\right)k_{ss}\right]H_{0}\;$$

where

(43) $$\;k_{i}=\left(\frac{{\left(\frac{b}{2}\right)}^{i}-1}{\frac{b}{2}-1}+{\left(\frac{b}{2}\right)}^{i-1}-1\right)H_{0}\;$$

(44) $$\;k_{i^{2}}=\left(\frac{{\left(\frac{b}{2}\right)}^{i^{2}}-1}{\frac{b}{2}-1}+{\left(\frac{b}{2}\right)}^{i^{2}-1}-1\right)H_{0}\;$$

(45) $$\;k_{ss}=\left(\frac{{\left(\frac{b}{2}\right)}^{s^{2}}-1}{\frac{b}{2}-1}+{\left(\frac{b}{2}\right)}^{s^{2}-1}-1\right)H_{0}\;$$

with *b* = 2 − *ρ*_0_.

**Figure 13:**
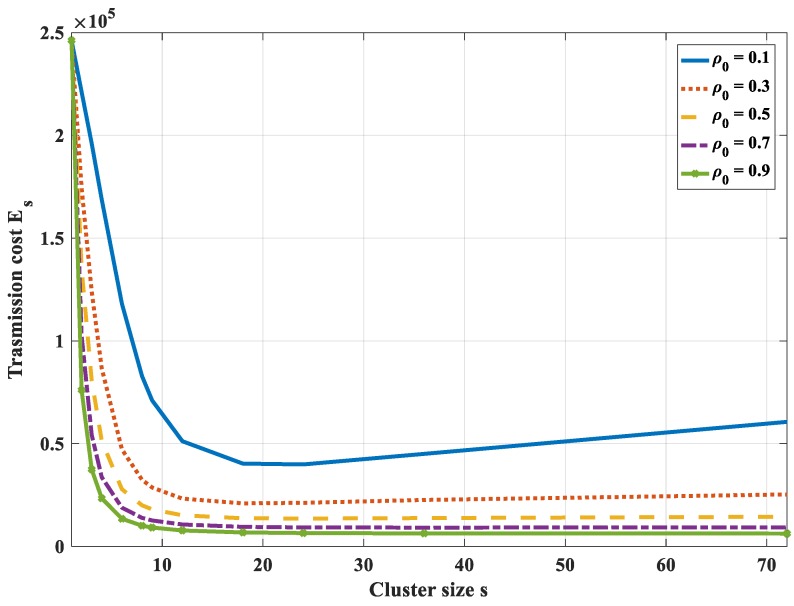
Total bit-hop cost *E_s_*, respectively, to cluster size with different values of entropy correlation coefficient in the case of 2-D with compression along SPT to the cluster head.

Figure 13 shows the total bit-hop cost *E_s_*, respectively, to cluster size with different values of *ρ*_0_ (in this case *n* = 72*, s* = [1, 2, 3, 4, 6, 8, 9, 12, 18, 24, 36, 72]). It is found that, with a small correlation coefficient value (*ρ*_0_ ≤ 0.3), there exists an optimal value of the cluster size, which is not at the two ends of the graph. However, in the case of the high correlation (*ρ*_0_ ≥ 0.5), the optimum number of the cluster size is *s_opt_* = *n*, i.e., it is not necessary to cluster the correlated region into smaller subsets. The reason is that, with high correlation, the energy for transmitting data from the cluster head to the sink becomes dominant. Then, the number of clusters should be reduced. Therefore, the cluster size becomes higher. Moreover, compression with a large number of highly correlated data is much better than dividing them into smaller groups.

b. Compression at the Cluster Head Only

In this case, at each cluster, a node receives data from the previous hop and transfers to the next hop without compression, which is shown in Figure 12b. The cluster head compressed all received data and sent it to the sink without any compression in the transfer path, which is shown in Figure 12c. The total bit-hop cost can be obtained as follows (detailed proof is in Appendix A.5).

(46) $$\;E_{s}\left(\rho_{0}\right)=\left[\frac{n^{2}}{6s}\left(s-1\right)\left(4s+1\right)+\frac{n}{6}\left(\frac{n}{s}-1\right)\left(4\frac{n}{s}+1\right)k_{ss}\right]H_{0}\;$$

where

$$\;k_{ss}=\left(\frac{{\left(\frac{b}{2}\right)}^{s^{2}}-1}{\frac{b}{2}-1}+{\left(\frac{b}{2}\right)}^{s^{2}-1}-1\right)H_{0}\;$$

with *b* = 2 − *ρ*_0_.

**Figure 14:**
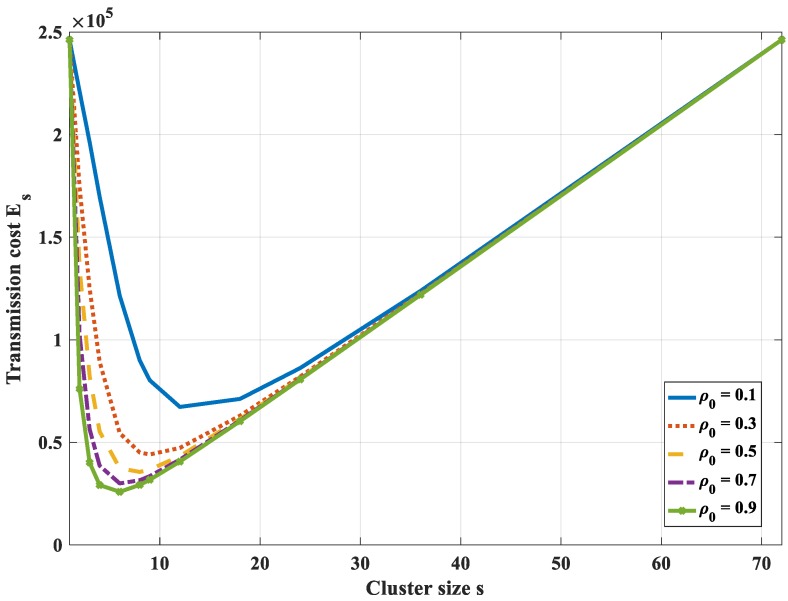
Total bit-hop cost *E_s_*, respectively, to cluster size with different values of the entropy correlation coefficient in the case of 2-D with compression at the cluster head only.

Figure 14 shows the total bit-hop cost *E_s_*, respectively, to cluster size with different values of *ρ*_0_ (in this case *n* = 72*, s* = [1, 2, 3, 4, 6, 8, 9, 12, 18, 24, 36, 72]). It is found that the optimal value of the cluster size is not at two ends. In this case, the transmission cost is highest at *s* = 1 and *s* = *n* because all nodes transfer data without compression. However, this optimal value depends on the correlation coefficient. With a higher correlation value, the optimal cluster size value is smaller.

#### 4.1.5. General Topology Model Analysis

**Figure 15:**
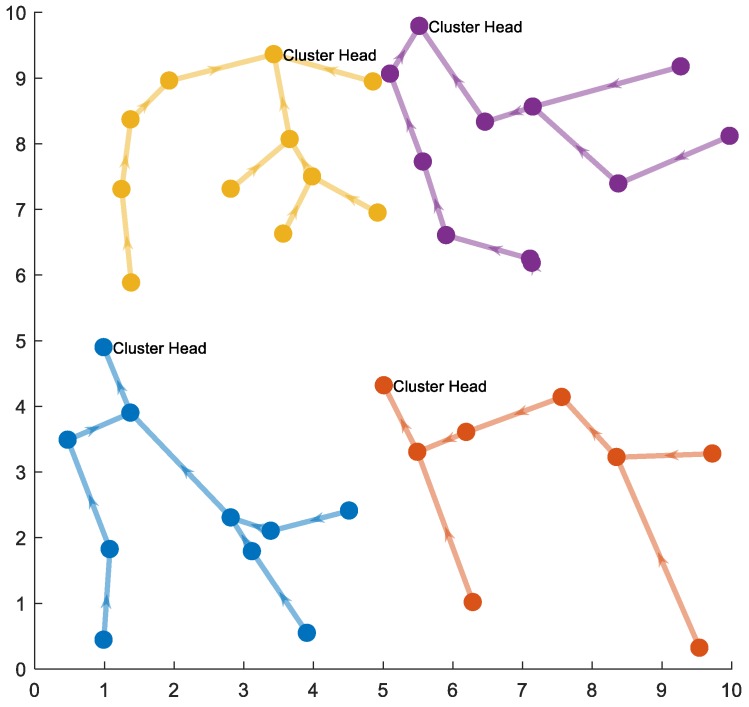
Illustration of clustering for a general topology model.

The analysis in the previous subsections is based on simple topology. To verify the robustness of our conclusions, we present the simulation with more general topology, which is shown in Figure 15.

Same as in Section 4.1.2, the network is assumed to be deployed in an *n* × *n* area. Nodes are deployed randomly over the network area. The network then can be divided into the correlated region with of size *s* × *s*. In this simulation, we choose the network size of 48 m *×* 48 m (*n* = 48), density of deployment is 1 node/m^2^, and *s* = [1, 2, 3, 4, 6, 8, 12, 16, 24, 48]*.* We also assumed that the energy consumption of clustering schemes compression is performed at intermediate nodes on SPT and only at the cluster heads. In addition, because the node position is randomly set, the distances between two nodes are also random values. Therefore, the bit-hop cost is not used. Instead, the transmission cost is calculated using the distance-based energy model, which is similar to Reference [33]. In this paper, for simplicity, the transmission cost is determined to be proportional to the square of the distance from the source to the destination.

a. Opportunistic Compression along the Short Path Tree to the Cluster Head

**Figure 16:**
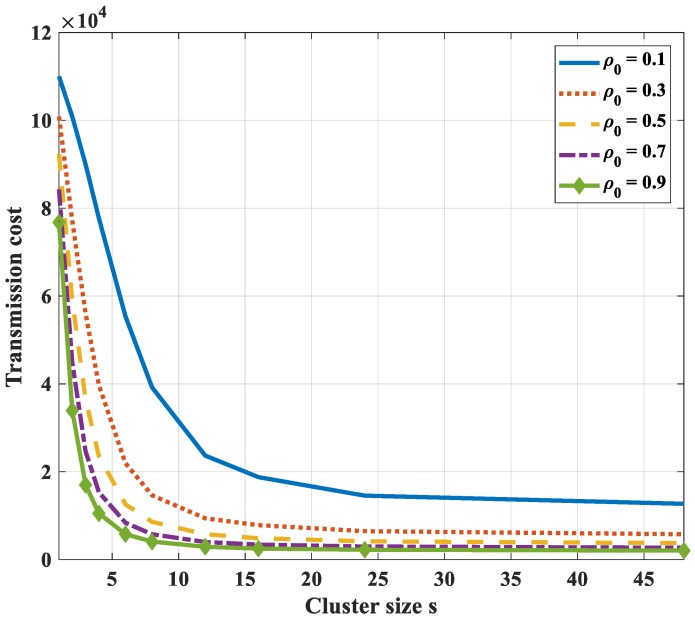
Total transmission cost, respectively, to cluster size with different values of an entropy correlation coefficient with compression along SPT to the cluster head.

In this case, each node transmits the amount of data that is equal to entropy/joint entropy of transmission data (including its own data and received data from other nodes, according to SPT). Entropy/joint entropy is calculated according to Equation (29) with *H*_0_ = 1. Figure 16 shows the total transmission cost, respectively, to cluster size with different values of *ρ*_0_. It is found that the simulation results are the same as our previous analysis. The optimal cluster size is *s* = *n*, i.e., all correlated regions should be a unique cluster.

b. Compression at the Cluster Head Only

**Figure 17:**
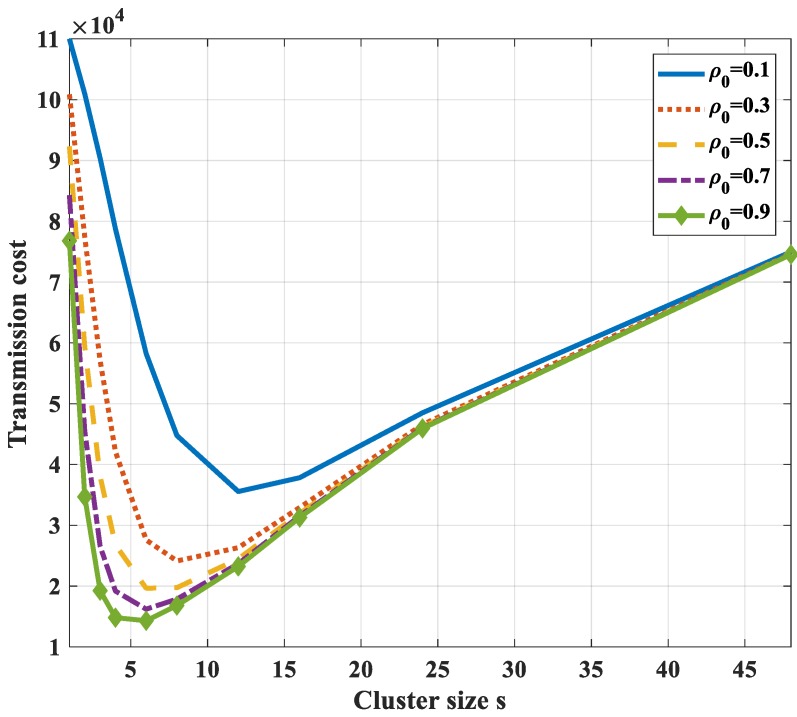
Total transmission cost, respectively, to cluster size with different values of the entropy correlation coefficient with compression at the cluster head only.

In this case, each node transmits its own data and transfers other data to the next node in SPT to the cluster head without compression. Compression is done only at the cluster head to send to the base station. Each node is assumed to have 1-bit own data. This Figure 17 the total transmission cost to cluster size with different values of *ρ*_0_. It is found that the simulation result is the same as our previous analysis. The optimal cluster size is not at two ends and this optimal value depends on the correlation coefficient. With a higher correlation value, the optimal cluster size value is smaller.

#### 4.1.6. Optimal Routing Schemes in Correlation Networks

In correlation networks, nodes are divided into correlated regions. From the above analyses with different topologies, the optimal routing scheme in correlation networks can be established below.

- In case compression along the transmission path to the cluster head is used, it is not necessary to divide a correlated region into smaller clusters to optimize the transmission cost. Instead, each correlated region becomes a cluster and the optimal routing path in each cluster is the shortest path from nodes to their cluster head.
- In the case of compression at the cluster head only and not at intermediate nodes, the transmission path is the shortest path to the cluster head. To get the optimal transmission cost, it is necessary to divide a correlated region into some smaller clusters. It is difficult to get the analytical solution of the optimal cluster size. Yet, we can draw the total transmission cost curves and find the nearly optimal value with a specified correlation coefficient and the number of network nodes.

In addition, the compression along the shortest path tree scheme have a lower transmission cost than compression only at the cluster head. It is obvious since the amount of transmitted data in the case of compression along the transmission path is smaller than compression at the cluster head only.

### 4.2. Representative Aggregation

In a correlated region with a high enough correlation level, it may not be necessary that every sensor node in a correlation group transmits its data to the sink. Instead, a smaller number of sensor measurements might be adequate to communicate the event features to the sink within a certain reliability level. These working sensors are called representative nodes of the region/group. To evaluate the reliability level, the distortion function is used.

#### 4.2.1. Distortion Function

The distortion function can be interpreted as the percentage of information loss due to the network resource constraints. This research considers the entropy correlation concept. Therefore, the predefined entropy distortion function proposed in Equation (29) is used.

Suppose that there are a total number of *N* sensor nodes in the considered area and denote their observed data as {*X*_1_, *X*_2_, …, *X_N_*}. The joint entropy of all these *N* sensors, *H*(*X*_1_, *X*_2_, …, *X_N_*), is the maximum amount of information that can be gained for the area of interest. If a subset of these sensors denoted as {*X_i_*_1_, *X_i_*_2_, …, *X_iM_*} are selected to report their observed data to the sink, the information gained at the sink is *H*(*X_i_*_1_, *X_i_*_2_, …, *X_iM_*). The distortion function is defined as the ratio of the decrease in the amount of information to the maximum amount of information, which is given by the equation below.

(47) $$\;D=\frac{H\left(X_{1},X_{2},\;\ldots ,\;X_{N}\right)-H\left(X_{i1},X_{i2},\;\ldots ,\;X_{iM}\right)}{H\left(X_{1},X_{2},\;\ldots ,\;X_{N}\right)}=1-\frac{H\left(X_{i1},X_{i2},\;\ldots ,\;X_{iM}\right)}{H\left(X_{1},X_{2},\;\ldots ,\;X_{N}\right)}\;$$

The value of *D* satisfies 0 ≤ *D* ≤ 1. Using the estimated joint entropy Equation (29), the distortion can be calculated by the formula below.

(48) $$\;D=1-\frac{k_{iM}}{k_{N}}\;$$

The distortion depends on the entropy correlation coefficient of the group, the number of representative nodes, and the total number of nodes in the group. If the desired distortion and the total number of nodes in the group is fixed, the number of representative nodes in a correlation group can be determined by using Equation (48).

#### 4.2.2. Number of Representative Nodes

As per the conclusion in the previous section, the number of representative nodes in a correlation group is determined based on an entropy correlation coefficient when the total number of nodes in the group is unchanged.

From Equation (48), we have formed the equation below.

(49) $$\;k_{iM}=\left(1-D\right)k_{N}\;$$

Moreover, using Equation (18), we have formed the formula below.

(50) $$\;k_{iM}=\frac{\beta^{iM}-1}{\beta -1}+\beta^{iM-1}-1\;$$

where

$$\;\beta =\frac{b}{2}=1-\frac{\rho }{2}\;$$

Then

(51) $$\;k_{iM}=\frac{\beta^{iM}-1}{\beta -1}+\frac{\beta^{iM}}{\beta }-1=\frac{\left(2\beta -1\right)\beta^{iM}-\beta^{2}}{\beta \left(\beta -1\right)}\;$$

This leads to

(52) $$\;iM=\log_{\beta }\left(\frac{\beta \left(\beta -1\right)k_{iM}+\beta^{2}}{2\beta -1}\right)\;$$

Substitute Equation (49) into Equation (51), we finally get the calculation of *iM* below.

(53) $$\;iM=\log_{\beta }\left(\frac{\beta \left(\beta -1\right)\left(1-D\right)k_{N}+\beta^{2}}{2\beta -1}\right)\;$$

in which *k_N_* is calculated using Equation (18).

Even though Equation (52) can be used to determine the number of representative nodes analytically, it is difficult to recognize the effect of correlation on the number of representative nodes. Thus, in this paper, a visual approach to estimate the number of representative nodes is presented.

Since the representative-based aggregation is more effective with the highly correlated region in this paper, the region with a high correlation level (*ρ*_0_ ≥ 0.5) is considered. In an environment with a high correlation level, the joint entropy will go to a “saturation” state when the number of nodes increases. Therefore, with the same distortion, the number of representative nodes does not depend on the number of total nodes in the group if the number of representative nodes is high enough. In the high correlation level (*ρ*_0_ ≥ 0.5), the estimated joint entropy goes to saturation when the number of nodes in the group reaches 14 nodes with distortion *D* ≈ 0, which is shown in Figure 3. For that reason, we only consider the total number less than 20 nodes (*N* ≤ 20).

**Figure 18:**
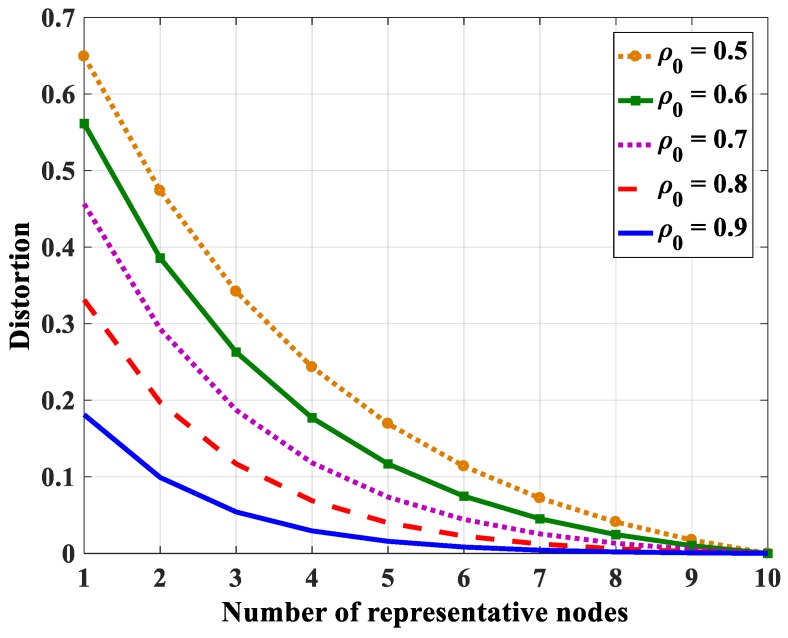
The relation between distortion and the number of representative nodes in the case *N* = 10.

**Figure 19:**
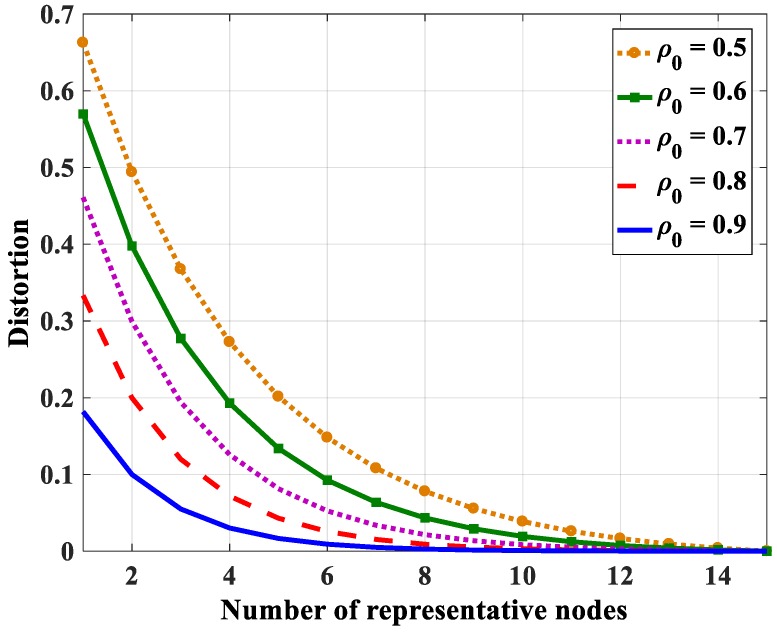
The relation between distortion and the number of representative nodes in the case *N* = 15.

**Figure 20:**
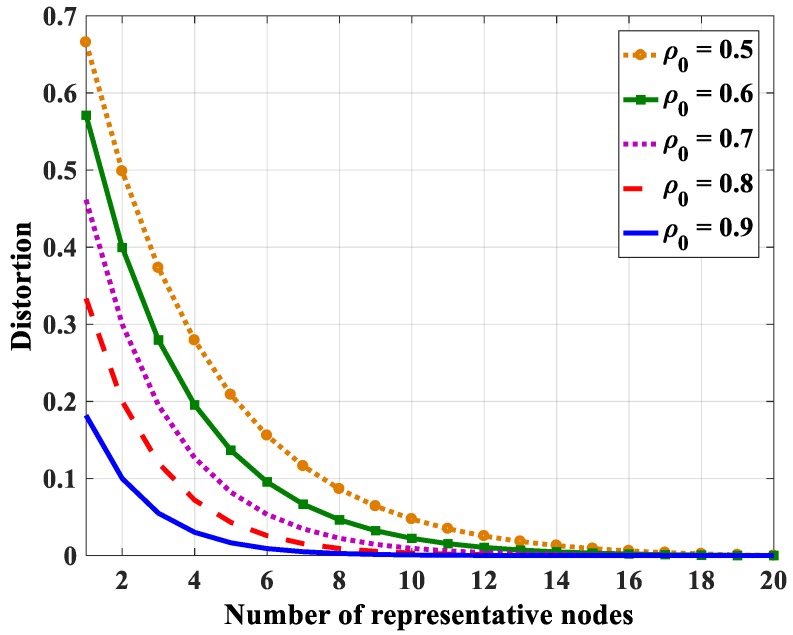
The relation between distortion and the number of representative nodes in the case *N* = 20.

Using Equation (48), the relations between the distortion and the number of representative nodes with different entropy correlation coefficients are shown in Figure 18 (in case *N* = 10), Figure 19 (in case *N* = 15), and Figure 20 (in case *N* = 20). The distortion becomes smaller when the entropy correlation coefficients as well as the number of representative nodes are higher. Specifically, three values of distortion (*D* = 0.05, 0.1, and 0.15) are chosen to determine the number of representative nodes with a different number of total nodes in the group. Table 5, Table 6 and Table 7 show the number of representative nodes in the cases of *D* = 0.05, 0.1, and 0.15, respectively. It can be found that, with the same distortion, the number of representative nodes is quite similar even though the number of nodes in a correlation group is different. It only depends on the entropy correlation coefficient, which is shown in Equation (52). The reason is that, in a correlational group, when the number of nodes is large enough, the joint entropy is almost unchanged. Thus, *k_N_* can be considered a constant number. For that reason, *iM* only depends on *β*, i.e., depends on the correlation coefficient.

It is noted that the above results are based on Equation (48) or Equation (52) with assumptions that the entropy of the nodes is similar, and the pairs of nodes have a similar entropy correlation coefficient. However, it is difficult to obtain these conditions practically. The selection of representative nodes, in some cases, may not satisfy the distortion requirement. Therefore, with the more practical assumption seen in Definition 2 of a correlated region, the value of the representative nodes should be increased by 1 node from the theoretical calculation to satisfy the distortion limit. This increment of representative nodes is found through practical experiences to guarantee the required distortion limit and make the selection of these nodes become more flexible. The detailed explanation will be shown in the practical validation section.

#### 4.2.3. Representative Node Selection

After knowing the number of representative nodes, it is necessary to select these nodes in the cluster group. The selection can be based on different purposes such as maximizing the total information (the obtained information from representative nodes is maximum), maximizing coverage (total covered areas by representative nodes is maximum), or energy balancing (the nodes with the highest remaining energy are chosen to be presentative nodes). In this paper, maximizing the obtained information from representative nodes in which the representative nodes are the least correlated ones when all other nodes in the group are chosen. This can be done by calculating the average entropy correlation coefficient of one node with all other nodes in the group and choose nodes with the least values of the average entropy correlation coefficient to be representative nodes. The selection algorithm of representative nodes by maximizing the obtained information is shown in Algorithm 2. 

The results of choosing representative nodes are presented in detail in the practical validation section below.

**Algorithm 2:** Maximizing the Obtained Information-Based Representative Node Selection Algorithm1. **BEGIN** 2. *C* = {*X*_1_, *X*_2_, …, *X_N_*};//*correlation node set* 3. **Initialize** new group *R* = *Φ*//*representative set* 4. **FOR**
*X_i_*, *X_j_ ∈ C* 5.  **Calculate**
$\rho \left(X_{i},X_{j}\right)$; 6.  **Calculate**
$\bar{\rho }\left(X_{i}\right)=\frac{1}{N}\overset{N}{\underset{j=1}{\sum }}\rho \left(X_{i},X_{j}\right)$; 7. **ENDFOR** 8. **FOR**
*k* = 1 to *iM* 9.  **Find**
$X_{i}=\arg\;\underset{X_{i}\in C}{\min}\left(\bar{\rho }\left(X_{i}\right)\right)$; 10.  **Add**
*X_i_* to *R*; 11.  **Remove**
*X_i_* from *C*; 12. **ENDFOR** 13. **END**

#### 4.2.4. Practical Validation

To validate the proposed representative node selection algorithm, we again use the temperature data supplied by the Intel Berkeley Research Lab [21]. In addition, the first correlation group with 11 nodes (dataset 1) is chosen at the same time as in the previous section.

At first, the number of representative nodes is chosen based on a theoretical calculation. In case the distortion is 0.05, the number of representative nodes is 8. By maximizing the obtained information, the selected representative nodes are {4, 8, 9, 15, 18, 21, 33, and 47}. The calculated distortion is 0.08 and does not satisfy the requirement that distortion must be less than or equal to 0.05. In the two other cases (*D* = 0.1 and *D* = 0.15), the actual distortions are still larger than the required distortion (see Table 8 for more details).

Next, the number of representative nodes is chosen based on a practical calculation (practical calculation equals the theoretical calculation plus one). Now, if we choose the distortion of 0.05, the number of representative nodes is 9. The selected representative nodes for maximizing the obtained information are {4, 8, 9, 15, 18, 21, 33, 42, and 47}. In this case, the real distortion is calculated as 0.05. In two other cases (*D* = 0.1 and *D* = 0.15), our selections are also satisfied with real distortions that are 0.1 and 0.15, respectively, which is shown in Table 9. In addition, the distortion limit requirement is always satisfied with all the possible selections of representative nodes.

For this dataset, it can be found that our selection is satisfied using our practical calculation and is unsatisfied with a theoretical calculation. The reason is because the chosen entropy range (Δ*H* = 0.4 = 20%*H*_0_) is quite large. This causes Equations (48) and (52) to become less precise.

Now we choose another dataset (dataset 2), also from the Intel Berkeley Research Lab [21], but at a different time. By using the proposed clustering algorithm, a set of 10 nodes, which are numbered by nodes {5, 21, 24, 31, 33, 40, 41, 42, 46, 47} are chosen. The entropy correlation coefficient is the same with the last dataset (*ρ*_0_ = 0.6) but the entropy range is smaller (Δ*H* = 0.3 = 12%*H*_0_). The detailed entropy values are shown in Table 10.

Similar to dataset 1, the number of representative nodes is calculated based on a theoretical calculation. In the case of the distortion being 0.05, the number of representative nodes is 7. By maximizing the obtained information, the selected representative nodes are {5, 24, 33, 40, 41, 42, and 46}. The calculated distortion is 0.068 and does not satisfy the requirement that distortion must be less than or equal to 0.05. However, in two other cases (*D* = 0.1 and *D* = 0.15), the actual distortions are equal to or smaller than the required distortion (see Table 11 for more details). As mentioned in the previous section, the theoretical selection of representative nodes, in some cases, may not be satisfied in practice especially with a small distortion.

Next, the number of representative nodes is chosen based on a practical calculation (practical calculation equals the theoretical calculation plus one). Now if the distortion is 0.05, the number of representative nodes is 8. The selected representative nodes for maximizing the obtained information are {5, 21, 24, 33, 40, 41, 42, and 46}. The actual distortion is 0.045, which satisfies the distortion. The distortion is less than or equal to 0.05. In two other cases (*D* = 0.1 and *D* = 0.15), our selections are always satisfied, which is shown in Table 12. In addition, the distortion limit requirement is always satisfied with all the possible selections of representative nodes.

From these two examples, it can be found that the selection of entropy range of nodes to establish the correlated region will affect the satisfaction of distortion. In our experiment, the maximum range should be 20% (Δ*H* = 20%*H*_0_) with a practical calculation, which is less than 10% with a theoretical calculation. It is recommended to use a practical calculation because the satisfied distortion will be obtained with not only maximum information selection but also maximum coverage, energy balance, or other selections.

## 5. Conclusions

The paper has proposed a novel practical correlation model to establish correlation regions using only the entropy of individual data and entropy correlation coefficients of data pairs. This model is built by looking at the data itself and it can overcome the limitation of other distance-dependent models. Moreover, the proposed model is simple to use because its arguments including node entropy and entropy correlation coefficients can be calculated from the real data with light computing efforts.

Using the proposed correlation model, the deployment of correlation characteristics can be analyzed in detail and the impacts of correlation to data aggregation can be evaluated thoroughly. For the compression aggregation in a high correlation environment (*ρ*_0_ ≥ 0.5) to obtain the optimal transmission cost, the network is clustered according to the correlated region. Each region corresponds to a cluster. In addition, in each cluster, data is transmitted and compressed along the shortest path to the cluster head.

For representative aggregation, using the estimated joint entropy model, the distortion function is established. Then the method to determine the number of representative nodes and the representative node selection algorithm is also proposed. The paper also analyzes the difference between the theoretical and practical aspect. To establish a correlation group and deploy the correlation characteristic, it is recommended to choose the large enough entropy correlation coefficient (*ρ*_0_ ≥ 0.5) and a small enough entropy range ((Δ*H ≤* 20% *H*_0_). In addition, the practical calculation of representative nodes should be used to obtain the flexible selection of the presentative nodes.

The paper has only considered fixed correlated regions, i.e., the correlation among nodes does not change along with time. In case correlated regions may change over time, it is necessary to have a mechanism to recognize the change. Then the network needs to be re-clustered according to new correlation relations. This will be considered carefully in the future and a complete routing protocol with data aggregation will be developed and implemented to validate our results in practice. Moreover, some other approaches that take advantage of correlation characteristics will be considered. In addition, changing the correlation group should also be considered to adapt to the change of sensed environments.

## Figures and Tables

**Table 1:** Node’s entropy of the dataset 1.

Node	4	5	8	9	15	18	21	23	33	42	47
**Entropy [bit]**	2.27	2.49	2.39	2.25	2.16	2.22	2.40	2.53	2.30	2.35	2.55

**Table 2:** Entropy correlation coefficient of each pair from the dataset 1.

Node	4	5	8	9	15	18	21	23	33	42	47
**4**	1	0.80	0.77	0.64	0.69	0.69	0.72	0.76	0.64	0.64	0.70
**5**	0.80	1	0.81	0.67	0.73	0.65	0.70	0.73	0.64	0.68	0.71
**8**	0.77	0.81	1	0.62	0.74	0.63	0.62	0.71	0.65	0.66	0.64
**9**	0.64	0.67	0.62	1	0.66	0.75	0.66	0.69	0.72	0.71	0.71
**15**	0.69	0.73	0.74	0.66	1	0.63	0.61	0.66	0.74	0.72	0.65
**18**	0.69	0.65	0.63	0.75	0.63	1	0.73	0.71	0.69	0.74	0.72
**21**	0.72	0.70	0.62	0.66	0.61	0.73	1	0.73	0.69	0.70	0.81
**23**	0.76	0.73	0.71	0.69	0.66	0.71	0.73	1	0.68	0.73	0.70
**33**	0.64	0.64	0.65	0.72	0.74	0.69	0.69	0.68	1	0.79	0.69
**42**	0.64	0.68	0.66	0.71	0.72	0.74	0.70	0.73	0.79	1	0.70
**47**	0.70	0.71	0.64	0.71	0.65	0.72	0.81	0.70	0.69	0.70	1

**Table 3:** Practical, upper bound and lower bound joint entropy (JE) of subsets from dataset 1.

Nodes in Subset	H_min_ [bit]	H_max_ [bit]	ρ_min_	ρ_max_	Practical JE [bit]	Upper Bound JE [bit]	Lower Bound JE [bit]
**4, 5**	2.27	2.49	0.80	0.80	2.84	2.99	2.72
**4, 5, 8**	2.27	2.49	0.77	0.81	3.13	3.41	2.96
**4, 5, 8, 9**	2.25	2.49	0.62	0.81	3.72	4.54	3.08
**4, 5, 8, 9, 15**	2.16	2.49	0.62	0.81	3.95	4.85	3.05
**4, 5, 8, 9, 15, 18**	2.16	2.49	0.62	0.81	4.22	5.06	3.10
**4, 5, 8, 9, 15, 18, 21**	2.16	2.49	0.61	0.81	4.51	5.21	3.13
**4, 5, 8, 9, 15, 18, 21, 23**	2.16	2.53	0.61	0.81	4.75	5.40	3.15
**4, 5, 8, 9, 15, 18, 21, 23, 33**	2.16	2.53	0.61	0.81	4.96	5.47	3.16
**4, 5, 8, 9, 15, 18, 21, 23, 33, 42**	2.16	2.53	0.61	0.81	5.06	5.52	3.16
**4, 5, 8, 9, 15, 18, 21, 23, 33, 42, 47**	2.16	2.55	0.61	0.81	5.23	5.60	3.17

**Table 4:** Clustering results of 48 nodes.

Group	Node Number	Entropy Range [bit]	*ρ*_0_
**1**	4, 5, 8, 9, 15, 18, 21, 23, 33, 42, 47	2.16–2.55	0.6
**2**	22, 24, 30, 31, 40, 41	2.64–3.09	0.6
**3**	1, 10, 11, 28, 35, 37, 39, 44, 46	1.83–2.17	0.5
**4**	2, 3, 6, 7, 12, 13, 14, 16, 17, 19, 20, 25, 26, 27, 29, 32, 34, 36, 38, 43, 45, 48	0.60–3.09	0.03

**Table 5:** Number of representative nodes with the distortion *D* = 0.05.

*ρ*_0_	0.5	0.6	0.7	0.8	0.9
*N* = 10	8	7	6	5	4
*N* = 15	10	8	7	5	4
*N* = 20	10	8	7	5	4

**Table 6:** Number of representative nodes with the distortion *D* = 0.1.

*ρ*_0_	0.5	0.6	0.7	0.8	0.9
*N* = 10	7	6	5	4	2
*N* = 15	8	6	5	4	2
*N* = 20	8	6	5	4	2

**Table 7:** Number of representative nodes with distortion *D* = 0.15.

*ρ*_0_	0.5	0.6	0.7	0.8	0.9
*N* = 10	6	5	4	3	2
*N* = 15	6	5	4	3	2
*N* = 20	7	5	4	3	2

**Table 8:** Selection of representative nodes and the actual distortion based on the theoretical calculation (dataset 1 with *N* = 11 nodes).

Desired Distortion	0.05	0.1	0.15
Number of representative nodes	8	6	5
Selected nodes	4, 8, 9, 15, 18, 21, 33, 47	8, 9, 15, 18, 21, 33	8, 9, 15, 18, 33
Actual distortion	0.08	0.15	0.20

**Table 9:** Selection of representative nodes and the actual distortion based on a practical calculation (dataset 1 with *N* = 11 nodes).

Desired Distortion	0.05	0.1	0.15
Number of Representative Nodes	9	7	6
Selected Nodes	4, 8, 9, 15, 18, 21, 33, 42, 47	8, 9, 15, 18, 21, 33, 47	8, 9, 15, 18, 21, 33
Actual Distortion	0.05	0.10	0.15

**Table 10:** Entropy values of 10 nodes in the correlated region (dataset 2 with *N* = 10 nodes).

Node Number	5	21	24	31	33	40	41	42	46	47
Entropy [bits]	2.79	2.74	2.64	2.84	2.80	2.81	2.68	2.62	2.81	2.82

**Table 11:** The selection of representative nodes and the actual distortion based on a theoretical calculation (dataset 2 with *N* = 10 nodes).

Desired Distortion	0.05	0.1	0.15
Number of Representative Nodes	7	6	5
Selected Nodes	5, 24, 33, 40, 41, 42, 46	5, 33, 40, 41, 42, 46	5, 40, 41, 42, 46
Actual Distortion	0.068	0.100	0.135

**Table 12:** The selection of representative nodes and the actual distortion based on a practical calculation (dataset 2 with *N* = 10 nodes).

Desired Distortion	0.05	0.1	0.15
Number of Representative Nodes	8	7	6
Selected Nodes	5, 24, 31, 33, 40, 41, 42, 46	5, 24, 33, 40, 41, 42, 46	5, 33, 40, 41, 42, 46
Actual Distortion	0.045	0.068	0.100

## Appendix A

### Appendix A.1. Proof that 0≤ρ≤1

According to Equation (3),

$$\;H\left(X,Y\right)\leq H\left(X\right)+H\left(Y\right)\Rightarrow \frac{H\left(X,Y\right)}{H\left(X\right)+H\left(Y\right)}\leq 1\;$$

In addition,

 H(X,Y)=H(X)+H(Y|X)≥H(X) 

Additionally,

 H(X,Y)=H(Y)+H(X|Y)≥H(Y) 

Then,

$$\;2H\left(X,Y\right)\geq H\left(X\right)+H\left(Y\right)\Rightarrow \frac{H\left(X,Y\right)}{H\left(X\right)+H\left(Y\right)}\geq \frac{1}{2}\;$$

Therefore,

$$\;0\leq 2-2\frac{H\left(X,Y\right)}{H\left(X\right)+H\left(Y\right)}\leq 1\;$$

Or

(A1) 0≤ρ(X,Y)≤1 

### Appendix A.2. Proof of Equation *(*38*)*

$$\begin{array}{r} E_{1in}=\overset{s-1}{\underset{i=1}{\sum }}H_{i}=H_{0}\overset{s-1}{\underset{i=1}{\sum }}k_{i}=H_{0}\;\left(\overset{s-1}{\underset{i=2}{\sum }}\frac{b}{2}(k_{i-1}+1)+1\right)\\ =H_{0}\left\{\frac{b}{2}\left(\overset{s-2}{\underset{i=1}{\sum }}k_{i}-k_{s-1}\right)+\frac{b}{2}\left(s-2\right)+1\right\}\; \end{array}$$

$$\begin{array}{r} E_{1in}=\frac{b}{2}H_{0}\overset{s-1}{\underset{i=1}{\sum }}k_{i}-\frac{b}{2}H_{0}k_{s-1}+H_{0}\left(\frac{b}{2}\left(s-2\right)+1\right)\\ =\frac{b}{2}E_{in}+H_{0}\left(\frac{b}{2}\left(s-1-k_{s-1}\right)+1-\frac{b}{2}\right) \end{array}$$

(A2) $$\;\Rightarrow E_{1in}=H_{0}\left(\frac{b}{2}\left(s-1-k_{s-1}\right)+1-\frac{b}{2}\right)/\left(1-\frac{b}{2}\right)=\left(\frac{b}{2-b}\left(s-1-k_{s-1}\right)+1\right)H_{0}\;$$

In addition,

(A3) $$\;E_{1ex}=P\cdot H_{s}=P\cdot k_{s}\cdot H_{0}\;$$

Therefore,

(A4) $$\;E_{s}\left(\rho_{0}\right)=\frac{N}{s}\left(E_{1in}+E_{1ex}\right)=\frac{N}{s}\left(\frac{b}{2-b}\left(s-1-k_{s-1}\right)+1+Pk_{s}\right)H_{0}\;$$

Moreover,

(A5) $$\;k_{s}=\frac{b}{2}\left(k_{s-1}+1\right)\Rightarrow k_{s-1}=\frac{2}{b}k_{s}-1\;$$

To replace (A4), we obtain

(A6) $$\begin{array}{r} E_{s}\left(\rho_{0}\right)=\frac{N}{s}\left(\frac{b}{2-b}\left(s-1-\left(\frac{2}{b}k_{s}-1\right)\right)+1+Pk_{s}\right)H_{0}\\ =\frac{N}{s}\left(\frac{b\cdot s}{2-b}+1+\left(P-\frac{2}{2-b}\right)k_{s}\right)H_{0}\; \end{array}$$

### Appendix A.3. Proof of Equation (40)

(A7) $$\;E_{1in}=\overset{s-1}{\underset{i=1}{\sum }}i\cdot H_{i}=\frac{s\left(s-1\right)}{2}H_{0}\;$$

In addition,

(A8) $$\;E_{1ex}=P\cdot H_{s}=P\cdot k_{s}\cdot H_{0}\;$$

Therefore,

(A9) $$\;E_{s}\left(\rho_{0}\right)=\frac{N}{s}\left(E_{1in}+E_{1ex}\right)=\frac{N}{s}\left(\frac{s\left(s-1\right)}{2}H_{0}+P\cdot k_{s}\cdot H_{0}\right)=N\left(\frac{s-1}{2}+\frac{P\cdot k_{s}}{s}\right)H_{0}\;$$

### Appendix A.4. Proof of Equation *(*42*)*

(A10) $$\;E_{2in}={\left(\frac{n}{s}\right)}^{2}\overset{s-1}{\underset{i=1}{\sum }}\left(2\left(s-i\right)H_{i}+H_{i^{2}}\right)={\left(\frac{n}{s}\right)}^{2}\left(\overset{s-1}{\underset{i=1}{\sum }}\left(2\left(s-i\right)k_{i}+k_{i^{2}}\right)\right)H_{0}\;$$

where

$$\;k_{i}=\left(\frac{{\left(\frac{b}{2}\right)}^{i}-1}{\frac{b}{2}-1}+{\left(\frac{b}{2}\right)}^{i-1}-1\right)H_{0}\;$$

$$\;k_{i^{2}}=\left(\frac{{\left(\frac{b}{2}\right)}^{i^{2}}-1}{\frac{b}{2}-1}+{\left(\frac{b}{2}\right)}^{i^{2}-1}-1\right)H_{0}\;$$

with *b* = 2 − *ρ*_0_.

In addition,

(A11) $$\;E_{2ex}=s\left(\overset{\frac{n}{s}-1}{\underset{i=1}{\sum }}2\left(\frac{n}{s}-i\right)i+i^{2}\right)H_{ss}=s\left(\overset{\frac{n}{s}-1}{\underset{i=1}{\sum }}2\left(\frac{n}{s}-i\right)i+i^{2}\right)k_{ss}H_{0}\;$$

where

$$\;k_{ss}=\frac{{\left(\frac{b}{2}\right)}^{s^{2}}-1}{\frac{b}{2}-1}+{\left(\frac{b}{2}\right)}^{s^{2}-1}-1\;$$

with *b* = 2 − *ρ*_0_.

We have

$$\begin{array}{cc} \overset{\frac{n}{s}-1}{\underset{i=1}{\sum }}2\left(\frac{n}{s}-i\right)i+i^{2} & =2\frac{n}{s}\overset{\frac{n}{s}-1}{\underset{i=1}{\sum }}i-\overset{\frac{n}{s}-1}{\underset{i=1}{\sum }}i^{2}={\left(\frac{n}{s}\right)}^{2}\left(\frac{n}{s}-1\right)-\frac{1}{6}\left(\frac{n}{s}-1\right)\left(\frac{n}{s}\right)\left(2\frac{n}{s}-1\right)\\ & =\frac{n}{6s}\left(\frac{n}{s}-1\right)\left(4\frac{n}{s}+1\right) \end{array}$$

Then,

$$\;E_{2ex}=\frac{n}{6}\left(\frac{n}{s}-1\right)\left(4\frac{n}{s}+1\right)H_{ss}=\frac{n}{6}\left(\frac{n}{s}-1\right)\left(4\frac{n}{s}+1\right)k_{ss}H_{0}\;$$

Lastly,

$$\begin{array}{l} E_{s}\left(\rho_{0}\right)=E_{2in}+E_{2ex}\\ \;={\left(\frac{n}{s}\right)}^{2}\left(\overset{s-1}{\underset{i=1}{\sum }}\left(2\left(s-i\right)k_{i}+k_{i^{2}}\right)\right)H_{0}+\frac{n}{6}\left(\frac{n}{s}-1\right)\left(4\frac{n}{s}+1\right)k_{ss}H_{0}\; \end{array}$$

(A12) $$\;E_{s}\left(\rho_{0}\right)=\left[{\left(\frac{n}{s}\right)}^{2}\overset{s-1}{\underset{i=1}{\sum }}\left(2\left(s-i\right)k_{i}+k_{i^{2}}\right)+\frac{n}{6}\left(\frac{n}{s}-1\right)\left(4\frac{n}{s}+1\right)k_{ss}\right]H_{0}\;$$

where

$$\;k_{i}=\left(\frac{{\left(\frac{b}{2}\right)}^{i}-1}{\frac{b}{2}-1}+{\left(\frac{b}{2}\right)}^{i-1}-1\right)H_{0}\;$$

$$\;k_{i^{2}}=\left(\frac{{\left(\frac{b}{2}\right)}^{i^{2}}-1}{\frac{b}{2}-1}+{\left(\frac{b}{2}\right)}^{i^{2}-1}-1\right)H_{0}\;$$

$$\;k_{ss}=\left(\frac{{\left(\frac{b}{2}\right)}^{s^{2}}-1}{\frac{b}{2}-1}+{\left(\frac{b}{2}\right)}^{s^{2}-1}-1\right)H_{0}\;$$

with *b* = 2 − *ρ*_0_.

### Appendix A.5. Proof of Equation *(*46*)*

$$\begin{array}{c} \;E_{2in}={\left(\frac{n}{s}\right)}^{2}\left(\overset{s-1}{\underset{i=1}{\sum }}2\left(s-i\right)i+i^{2}\right)H_{0}={\left(\frac{n}{s}\right)}^{2}\left(2s\overset{s-1}{\underset{i=1}{\sum }}i-\overset{s-1}{\underset{i=1}{\sum }}i^{2}\right)H_{0}\\ ={\left(\frac{n}{s}\right)}^{2}\left(\frac{2s^{2}\left(s-1\right)}{2}-\frac{s\left(s-1\right)\left(2s-1\right)}{6}\right)H_{0}\; \end{array}$$

(A13) $$\;E_{2in}={\left(\frac{n}{s}\right)}^{2}\frac{s\left(s-1\right)\left(4s+1\right)}{6}H_{0}=\frac{n^{2}}{6s}\left(s-1\right)\left(4s+1\right)H_{0}\;$$

In addition,

$$\;E_{2ex}=s\left(\overset{\frac{n}{s}-1}{\underset{i=1}{\sum }}2\left(\frac{n}{s}-i\right)i+i^{2}\right)H_{ss}=\frac{n}{6}\left(\frac{n}{s}-1\right)\left(4\frac{n}{s}+1\right)H_{ss}\;$$

(A14) $$\;E_{2ex}=\frac{n}{6}\left(\frac{n}{s}-1\right)\left(4\frac{n}{s}+1\right)k_{ss}H_{0}\;$$

Then,

$$\;E_{s}\left(\rho_{0}\right)=E_{2in}+E_{2ex}=\frac{n^{2}}{6s}\left(s-1\right)\left(4s+1\right)H_{0}+\frac{n}{6}\left(\frac{n}{s}-1\right)\left(4\frac{n}{s}+1\right)k_{ss}H_{0}\;$$

(A15) $$\;E_{s}\left(\rho_{0}\right)=\left[\frac{n^{2}}{6s}\left(s-1\right)\left(4s+1\right)+\frac{n}{6}\left(\frac{n}{s}-1\right)\left(4\frac{n}{s}+1\right)k_{ss}\right]H_{0}\;$$

where

$$k_{ss}=\left(\frac{{\left(\frac{b}{2}\right)}^{s^{2}}-1}{\frac{b}{2}-1}+{\left(\frac{b}{2}\right)}^{s^{2}-1}-1\right)H_{0}\;\mathrm{with}\;b\;=\;2-\rho_{0}$$
